# Evolutionarily Divergent, Unstable Filamentous Actin Is Essential for Gliding Motility in Apicomplexan Parasites

**DOI:** 10.1371/journal.ppat.1002280

**Published:** 2011-10-06

**Authors:** Kristen M. Skillman, Karthikeyan Diraviyam, Asis Khan, Keliang Tang, David Sept, L. David Sibley

**Affiliations:** 1 Department of Molecular Microbiology, Washington University School of Medicine, St. Louis, Missouri; 2 Department of Biomedical Engineering and Center for Computational Medicine and Bioinformatics, University of Michigan, Ann Arbor, Michigan; University of Georgia, United States of America

## Abstract

Apicomplexan parasites rely on a novel form of actin-based motility called gliding, which depends on parasite actin polymerization, to migrate through their hosts and invade cells. However, parasite actins are divergent both in sequence and function and only form short, unstable filaments in contrast to the stability of conventional actin filaments. The molecular basis for parasite actin filament instability and its relationship to gliding motility remain unresolved. We demonstrate that recombinant *Toxoplasma* (TgACTI) and *Plasmodium* (PfACTI and PfACTII) actins polymerized into very short filaments *in vitro* but were induced to form long, stable filaments by addition of equimolar levels of phalloidin. Parasite actins contain a conserved phalloidin-binding site as determined by molecular modeling and computational docking, yet vary in several residues that are predicted to impact filament stability. In particular, two residues were identified that form intermolecular contacts between different protomers in conventional actin filaments and these residues showed non-conservative differences in apicomplexan parasites. Substitution of divergent residues found in TgACTI with those from mammalian actin resulted in formation of longer, more stable filaments *in vitro*. Expression of these stabilized actins in *T. gondii* increased sensitivity to the actin-stabilizing compound jasplakinolide and disrupted normal gliding motility in the absence of treatment. These results identify the molecular basis for short, dynamic filaments in apicomplexan parasites and demonstrate that inherent instability of parasite actin filaments is a critical adaptation for gliding motility.

## Author Summary

Cellular movement is key to life and in the case of intracellular parasites, provides a vital mechanism to gain access to the protected niche they require. The parasite *Toxoplasma gondii* is a model for a group of parasites called apicomplexans, which move by an actin-dependent process referred to as gliding motility. This form of motility is distinct from that used by ciliated or flagellated cells, and from the crawling behavior of amoeba and many mammalian cells. We demonstrate that the normally highly conserved protein actin is divergent in these parasites and that it displays unusual kinetic properties that result in formation of short unstable filaments, in contrast to the highly stable nature of mammalian actin. Our findings reveal that the short dynamic nature of parasite actins is due to a small number of amino acid differences that affect stability of the filament. Moreover, these properties are essential to normal parasite motility since reversion of these residues to match those seen in mammalian cells was detrimental to gliding movement. The dependence of parasites on rapid turnover of highly unstable actins renders them extremely sensitive to toxins that stabilize actin filaments, thus providing a potential target for development of specific intervention.

## Introduction

Actin is an essential protein that is highly conserved in sequence and function in eukaryotic cells. Despite this conservation, parasites within the phylum Apicomplexa encode divergent actins that remain largely in an unpolymerized state *in vivo* and only form short, unstable filaments *in vitro*, in contrast to conventional actins from yeast to mammals. Apicomplexan parasites are obligate intracellular protozoan pathogens of animals including humans. Two notable members of this phylum are *Toxoplasma gondii* [1], an opportunistic pathogen, and *Plasmodium falciparum*, the most severe cause of malaria [2], a devastating global disease. Apicomplexan parasites move by a unique form of gliding motility that is actin-dependent [3]. Initial studies demonstrated that host cell invasion by *T. gondii* [4] is blocked by cytochalasin D, and it was later shown using a combination of genetic mutants in the host *vs.* parasite that the primary target of these treatments was parasite actin filaments, which are essential for motility and cell invasion [5]. Gliding motility is considered to be a conserved feature of the phylum [6] and has been described in *T. gondii* tachyzoites [7], *Plasmodium* spp. sporozoites [8], *Cryptosporidium* spp. sporozoites [9], *Eimeria* sporozoites [10] and the more distantly related gregarines [11]. Gliding motility powers migration through tissues, traversal of biological barriers, and invasion into and egress from host cells [12].

*T. gondii* contains a single actin gene, TgACTI, which shows 83% amino acid identity with mammalian muscle actin [13]. *P. falciparum* contains two actin genes, PfACTI, that is closely related to TgACTI, sharing 93% identity at the protein level, and PfACTII, which is more divergent and has only 79% similarity to PfACTI [14]. Transcriptional analysis demonstrates that PfACTI is expressed throughout the parasite life cycle while PfACTII is most highly expressed in gametocytes [15]. Parasite actins have been shown to exist mostly in an unpolymerized state, as defined by sedimentation at 100,000*g* and an absence of staining in fixed cells with fluorescently labeled phalloidin [16], [17]. In contrast, the majority of actin in mammalian, yeast, and amoeba cells is found in long filamentous networks, or bundled fibers, which are readily stained with phalloidin and sedimented by centrifugation at 100,000*g* [18]. Although very uncommon in apicomplexans, actin filaments have been visualized by freeze fracture electron microscopy beneath the parasite membrane in gliding *T. gondii* tachyzoites [17]. PfACTI from *P. falciparum* has been shown to form short filaments *in vitro* [19], and similar short filaments of ∼100 nm in length were detected in lysates from asexually propagating stages (i.e. merozoites) following sedimentation at 500,000*g* [16]. Apicomplexans are also unusual in having a streamlined set of actin binding proteins consisting of actin depolymerizing factor, cyclase associated protein, profilin, and capping protein [20], [21], while they lack Arp2/3 [22] and many other regulatory proteins found in more complex systems.

Actin dynamics are controlled in part by an inherent ability of actin monomers to polymerize head-to-tail into parallel helical strands that form filaments [18]. Polymerization is dependent on Mg^2+^, salt (i.e. KCl), and ATP-actin and is thermodynamically favored above the so-called critical concentration (Cc) [18]. Above the Cc, filaments are typically highly stable, although gradual hydrolysis of ATP and release of P_i_ increases disassembly and susceptibility to severing [18]. TgACTI also requires high salt and Mg^2+^ for polymerization, and somewhat surprisingly it initiates polymerization more readily than conventional actins, and yet it only forms short transient filaments *in vivo* [23]. Consistent with this finding, *in vitro* polymerization of TgACTI results in formation of short, irregular filaments that rapidly disassemble in the absence of stabilizing compounds such as phalloidin [23]. TgACTI fails to copolymerize with mammalian actin [23]; however, copolymerization of PfACTI with rabbit muscle actin reveals differences in monomer stacking and a larger helical pitch in parasite actin [24]. Consistent with this, previous modeling studies have suggested that instability of parasite actin filaments might arise from structural changes [23], although this hypothesis has not been directly tested.

Highly motile cells often exhibit rapid actin turnover [18], suggesting that the unusual dynamics of apicomplexan actins may be important in gliding motility. Indirect evidence that actin turnover is important in *T. gondii* comes from treatment with agents that stabilize actin filaments, such as the heterocyclic compound jasplakinolide (JAS), which is produced by marine sponges and acts to stabilize actin filaments [25]. JAS treatment disrupts motility and cell invasion in *T. gondii* [17], [26], as well as invasion of merozoites [27], motility of ookinetes [28], and endocytic trafficking in trophozoites [29] of *Plasmodium*. Collectively, previous studies indicate that apicomplexan actins spontaneously polymerize into short filaments that are intrinsically unstable; however, the molecular basis and functional significance of these unusual properties are largely unknown.

The present study was undertaken to address two questions: 1) what intrinsic properties govern actin filament instability in apicomplexans?, and 2) are the unusual dynamic properties of filamentous actin important for efficient motility in apicomplexans? Here, we demonstrate that two divergent residues partially explain the inherent instability of parasite actin filaments and reveal that this feature is important for efficient gliding motility in *T. gondii*.

## Results

### Apicomplexan actin filaments are inherently unstable and rescued by phalloidin

**Figure 1:**
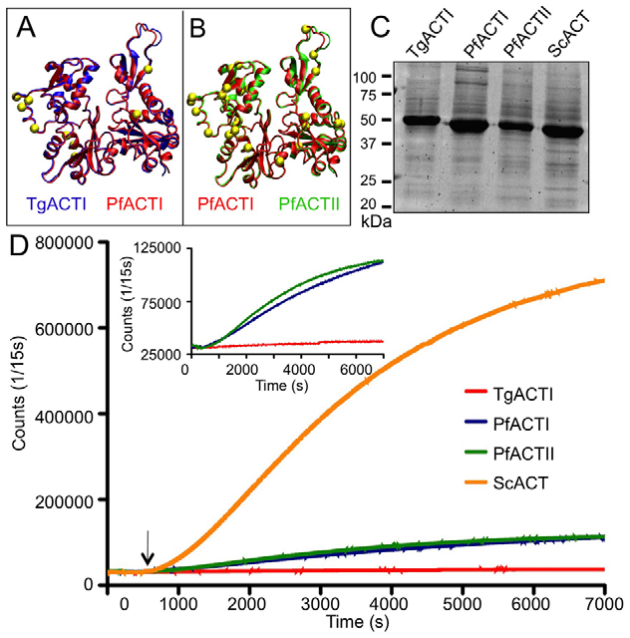
Apicomplexan actins differ in sequence and polymerization kinetics. (A) Model of TgACTI (blue) mapped onto PfACTI (red) highlighting amino acid differences (yellow). (B) Model of PfACTI (red) mapped onto PfACTII (green) highlighting amino acid differences (yellow). (See supplementary **Figure S6** for enlargement). (C) Expression of recombinant apicomplexan actins purified from baculovirus, resolved using a 12% SDS-PAGE gel, stained with SYPRO Ruby. (D) Comparison of actin polymerization kinetics. Polymerization of 5 µM actin was induced by the addition of F buffer (arrow) and monitored by light scattering. Insert shows parasite actins on the expanded Y-axis.

Despite overall conservation in sequence, apicomplexan actins are functionally divergent from actins in yeast, animals, and plants; likely due to molecular differences in parasite actins that affect function (see supplemental **Figure S1**). To visualize these shared differences, homology models were created to compare parasite actins: TgACTI and PfACTI are highly similar (**Figure 1A**, yellow spheres highlight differences) while PfACTII is more divergent (**Figure 1B**). We expressed recombinant actins from *T. gondii* (TgACTI), *P. falciparum* (PfACTI, PfACTII), and yeast (ScACT) using baculovirus and purified these proteins to study their properties *in vitro*, as described previously [23] (**Figure 1C**). Recombinant actins contained an N-terminal His_6_ tag that was used for purification; previous studies have shown that the presence of this tag does not alter polymerization [23]. The kinetics of actin polymerization was examined by light scattering following addition of filamentation (F) buffer. TgACTI only polymerized to a very limited extent (**Figure 1D**, red), while both PfACTI and PfACTII showed modest levels of polymerization (**Figure 1D**, blue and green, respectively). In contrast, polymerization of ScACT (**Figure 1D**, orange) was much more efficient, indicating that the inefficiency of parasite actin polymerization was not a consequence of expression in baculovirus, or the N-terminal tag shared by all of the proteins.

**Figure 2:**
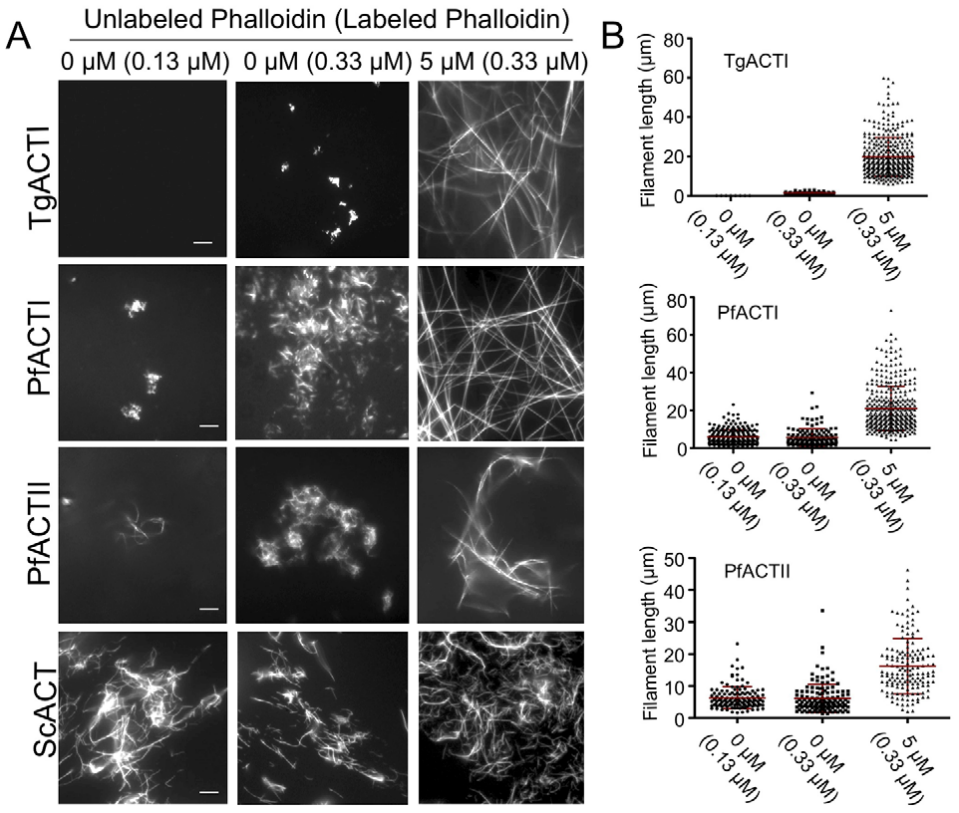
Apicomplexan actins form inherently unstable filaments that are rescued by phalloidin. (A) *In vitro* polymerization of parasite actins visualized by fluorescence microscopy of phalloidin stained actin. Parasite actins were incubated at 5 µM with no addition (0) or with equimolar unlabeled phalloidin (5 µM) and visualized by addition of low levels of Alexa-488 labeled phalloidin (0.13 µM or 0.33 µM) and visualized by fluorescence microscopy. The values at the top of the figure panel indicate the amounts of phalloidin used in each reaction: µM unlabeled phalloidin (µM labeled phalloidin). Scale bars, 5 µm. Representative of three or more similar experiments. (B) Quantitation of length of filaments formed during *in vitro* polymerization of parasite actins. Mean ± S.D. shown by horizontal line. PfACTI and PfACTII formed significantly longer filaments than TgACTI at either of the low doses of phalloidin added for visualization (*i.e.* 0.13 or 0.33 µM) (Student's *t*-test, *P*<0.001). Concentrations of phalloidin used for treatment are indicated on below the graphs as µM unlabeled phalloidin (µM labeled phalloidin).

To test the ability of parasite actins to polymerize under stabilizing conditions, purified actins were incubated with different amounts of phalloidin during polymerization in F buffer. Consistent with previous reports [26], [30], filaments were not detected for TgACTI in the presence of low levels of fluorescently labeled phalloidin (i.e. 0.13 µM) that was added to visualize filamentous actin (**Figure 2A**). Short, punctate filaments were observed when a slightly higher level of labeled phalloidin (i.e. 0.33 µM) was added to TgACTI (**Figure 2A,B**). In contrast, long clusters of filaments were observed when TgACTI was allowed to polymerize in the presence of equimolar levels of unlabeled phalloidin combined with lower levels of labeled phalloidin for visualization (i.e. 0.33 µM) (**Figure 2A,B**). A similar dose-response to increasing phalloidin was seen for PfACTI and PfACTII, although these actins also occasionally formed small clusters of short filaments even in low levels of labeled phalloidin (i.e. 0.13 µM) (although rare, a representative example is shown in **Figure 2A,B**). Both PfACTI and PfACTII formed more abundant clusters of short filaments in slightly higher levels of labeled phalloidin (i.e. 0.33 µM) and these were further stabilized by equimolar unlabeled phalloidin (**Figure 2A,B**). As expected, ScACT formed long, stable filaments regardless of the phalloidin concentration (**Figure 2A**). Interestingly, the filaments formed by ScACT and PfACTII in the presence of high levels of phalloidin (equimolar) were often curved, while those of TgACTI and PfACTI where extremely straight (**Figure 2**). Measurement of the sizes of individual filaments formed by these different actins in response to phalloidin confirmed the general patterns seen by microscopy. Both PfACTI and PfACTII formed significantly longer filamentous structures than TgACTI in the presence of low levels of phalloidin used to visualize filaments (*i.e.* 0.13 or 0.33 µM), and all three actins showed a shift to longer filaments with with equimolar phalloidin treatment (**Figure 2B**).

**Figure 3 ppat-1002280-g003:**
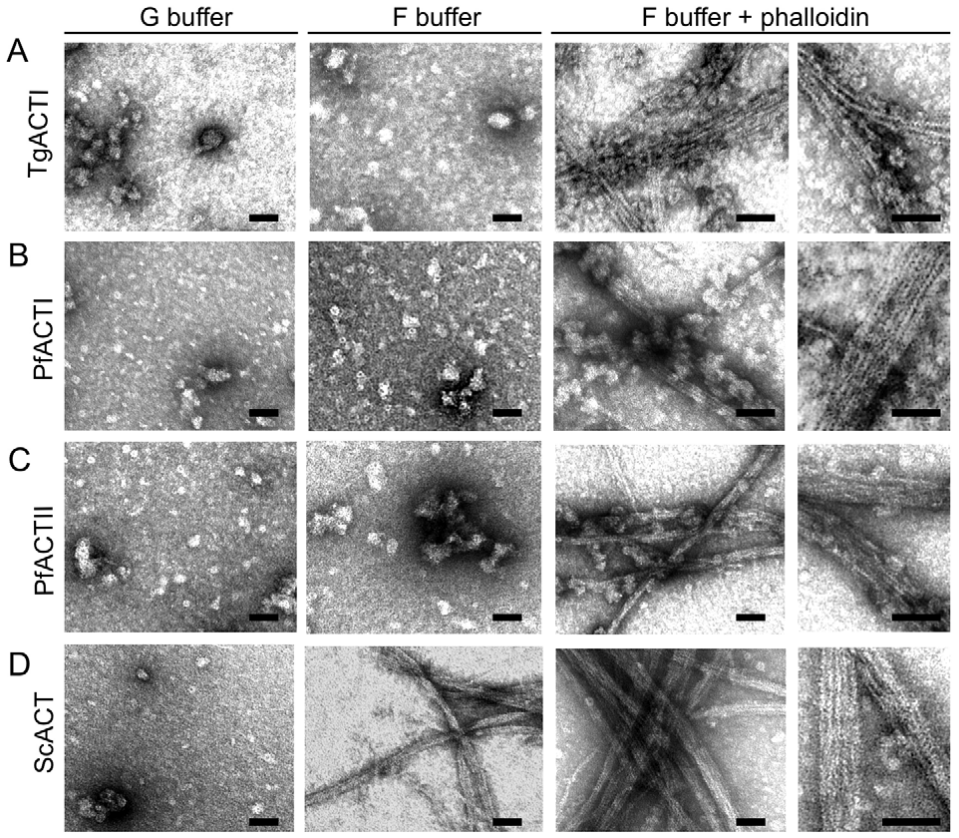
Ultrastructural features of parasite actins revealed by electron microscopy. (A) TgACTI was incubated in G buffer or F buffer with or without equimolar concentration of phalloidin for 1 hr. The reactions were added to grids, negatively stained with uranyl acetate and examined by EM. Identical conditions were used to observe PfACTI (B), PfACTII (C) and ScACT (D). Images are representative of 3 or more experiments. Scale bars, 50 nm.

Filaments formed by parasite actins were examined by negative staining and electron microscopy to reveal ultrastructural details. Similar to the fluorescent phalloidin assays, EM visualization of abundant parasite actin filaments required incubation in F buffer containing equimolar phalloidin (**Figure 3**). In the absence of added phalloidin, the parasite actins were observed to form irregular globular aggregates. Although we did not detect structures by EM that were similar to the small clusters seen by fluorescence staining of PfACTI and PfACTII in low levels of phalloidin (**Figure 2A**), this may reflect the low frequency of these forms or a requirement for low levels of phalloidin to stabilize them. Enlarged images of the phalloidin stabilized filaments formed by the three parasite actins revealed a spiral pattern of the actin helix and striations along the filament, which are typical characteristics of conventional actin filaments, as observed in ScACT filaments formed under all polymerizing conditions (**Figure 3**). Both the parasite actins and yeast actin showed prominent filament bundles, which are also seen in the fluorescence images mentioned above. Collectively, these studies verify that the instability of parasite actin filaments generated from recombinant tagged actins is intrinsic and that polymerization is rescued by high concentrations of phalloidin.

### Homology modeling of the phalloidin-binding site within parasite actins filaments

**Figure 4 ppat-1002280-g004:**
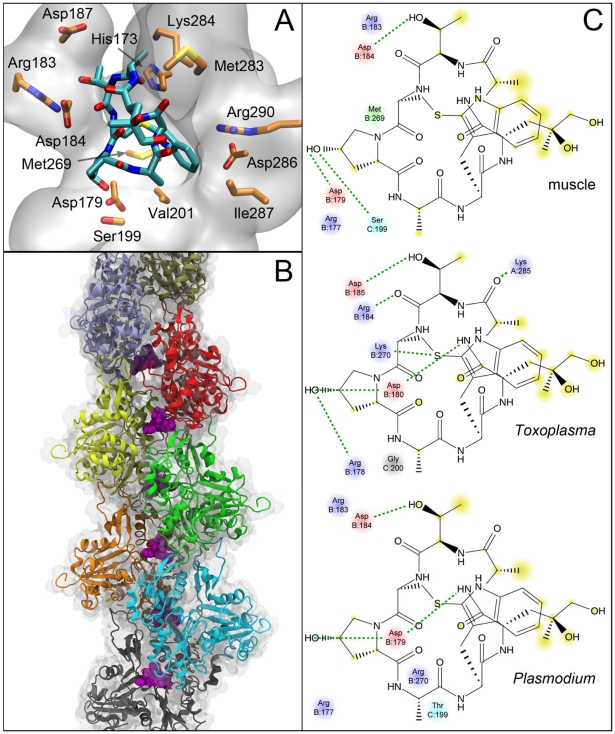
Predicted binding site of phalloidin in muscle and parasite actin filaments. Molecular details of the interaction of phalloidin in the muscle actin filament. (A) Side chains of amino acids within 3.5 Å are explicitly shown and the protein around phalloidin is depicted as a transparent surface. Hydrogens are omitted for clarity. (B) Position of phalloidin (purple) in the filament showing its interaction with three individual protomers. (C) 2D interaction diagrams showing the interaction differences between muscle, TgACTI and PfACTII actin. Hydrogen bonding interaction are depicted by dashed green lines and portions of the molecule that are solvent accessible are highlighted in yellow. Blue-basic residues; red-acidic residues; cyan-polar residues.

To investigate the molecular basis of phalloidin binding, we used structures from our molecular dynamics (MD) simulation of the muscle actin filament and performed molecular docking studies with phalloidin (**Figures 4A, 4B**). Our predicted phalloidin binding site is similar to that reported previously [31], but also provides more precise information on specific binding contacts that stem from the following improvements: 1) unconstrained docking analyses were based on a new higher resolution actin filament model [32]; 2) flexible protein conformations were included by choosing multiple snapshots from MD simulations and multiple binding sites were included within each snapshot; 3) induced fit was accommodated by simulated annealing. Together these analyses precisely mapped the phalloidin binding site in mammalian actin to the loop formed by residues 196–200 in the lower actin monomer, the 72–74 loop of the middle monomer, and the 285–290 loop of the upper monomer (**Figure 4A**). These three regions closely coincide with those identified in previous experimental studies as important for phalloidin interactions [31], [33], [34]. Importantly, residues including D179, Y198, S199, K284, I287 and R290, which were previously observed to be close to the phalloidin binding site [31], were also within 4 Å of phalloidin in our model. Moreover, our more precise placement of phalloidin predicts maximum interaction between the Cys3-Pro (OH)4-Ala5-Trp6 ring in phalloidin and actin residues, while Leu(OH)7 in phalloidin faces out of the binding pocket and is accessible to solvent. This orientation corresponds well with experimental studies [31], [33], [34] showing that derivatives of phalloidin with a fluorophore linked to Leu(OH)7, bind actin filaments in the same conformation.

Homology models for TgACTI and PfACTII were built using the muscle actin filament obtained by simulated annealing. Docking studies were repeated using TgACTI and PfACTII homology models and they yielded very similar conformations although the specific amino acid contacts lying within 4 Å varied slightly between proteins. Residues previously shown by mutational analysis to mediate phalloidin binding in yeast [35] (*i.e.* R177, D179), were conserved in all three models (**Figure 4C**). Residues R177 and D179 in mammalian actin, corresponding to R178 and D180 in parasite TgACTI, both lie within 4 Å of phalloidin (**Figure 4C**). Six specific differences in the residues contacting phalloidin in mammalian muscle actin *vs.* TgACTI and PfACTII were noted (**Figure 4C**, see supplemental **Figure S1**). Together, these differences may mediate the less efficient binding to phalloidin observed for parasite actin filaments.

### TgACTI filament stability is restored by substitution with conventional residues

**Figure 5:**
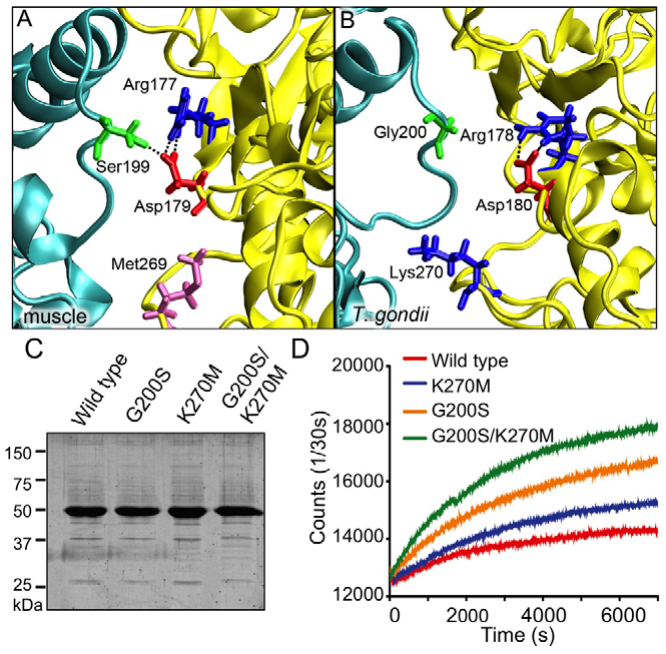
Identification of single substitutions within TgACTI that affect filament stability. (A) Modeling of S199-D179 hydrogen bond and M269 in the hydrophobic loop of mammalian actin (B) Modeling of loss of hydrogen bond with G200 substitution and reduced hydrophobicity at position 270 in TgACTI. (C) Expression of TgACTI recombinant proteins containing mammalian-like substitutions in baculovirus, resolved using a 12% SDS-PAGE gel, and stained with SYPRO Ruby. (D) Comparison of polymerization kinetics of TgACTI substitutions. F buffer was added at time = 0 sec to induce polymerization of 5 µM actin and polymerization was monitored by light scattering.

Molecular modeling was also used to identify residues that differ between human muscle actin and TgACTI in regions that are predicted to be critical for stabilizing the actin filament. Divergent residues at positions G200 and K270 in *T. gondii* were identified as candidates that likely affect monomer-monomer interactions across the filament. However, the previously identified difference R277 in TgACTI, corresponding to glutamate in muscle [23], no longer made close contact in the new filament model, and consistent with this, no change in polymerization of substituted TgACTI-R277E was observed (data not shown). Instead, our refined model now points to S199 in human muscle actin as forming an important hydrogen bond with D179 of a monomer across the filament (**Figure 5A**). This hydrogen bonding was observed in a majority of inter-monomer contacts predicted in the MD simulations of the filament. However, at this position TgACTI contains a glycine that would eliminate the hydrogen bond and potentially adversely impact filament stability (**Figure 5B**). The second residue of interest identified was M269 in muscle actin (**Figure 5A**) that corresponds to K270 in TgACTI (**Figure 5B**). Mutational studies in yeast have previously demonstrated that loss of hydrophobicity in this loop leads to destabilization of the actin filament [36], and it has previously been suggested that this natural difference may affect parasite actin stability [23].

**Figure 6:**
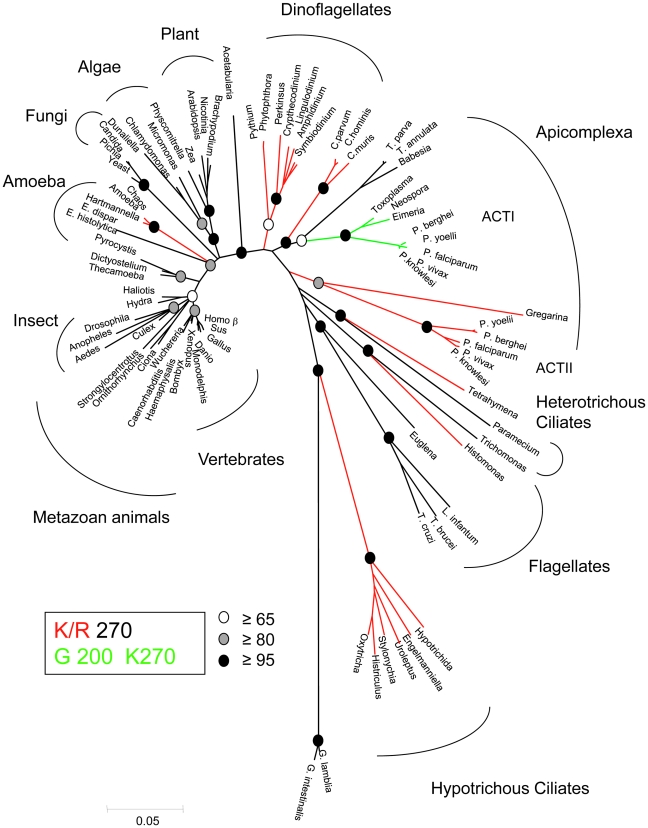
Phylogenetic tree highlighting the diversity of actins. Actin sequences were retrieved from Genbank and aligned using Clustal. Neighbor joining with PAUP* was used to assemble the sequences into phylogenetic tree based on 1000 bootstrap replicates (bootstrap values indicted in circles at specific nodes). The branches are color coded to show phylogenetic differences. Red represents branches containing a lysine or arginine at position 270, which is conventionally a methionine. Green represents branches that contain the K270 substitution as well as a glycine at position 200, which is conventionally a serine or threonine. Numbering based on the TgACTI sequence.

All three of the parasite actins studied here contain the alteration K/R270, whereas G200 is found in both TgACTI and PfACTI, while PfACTII has a threonine at this position (see supplemental **Figure S1**). In comparing other actins to those studied here, the alteration in G200 seen in *T. gondii* is conserved only in ACTI homologues found in a subset of apicomplexans that rely on gliding motility (**Figure 6**). In contrast, the substitution of K/R in the hydrophobic plug at residue 269/270 is seen in a wider variety of protozoa including dinoflagelates, ciliates, and apicomplexans (**Figure 6**). The distribution of these residues among taxa on the phylogenetic trees suggests a very different ancestry for these two alterations. The presence of a positive charged residue at 269/270 is polyphyletic, being found in a wide diversity of taxa, although not in higher plants or animals. In contrast, the G200, which was found in combination with K270, is confined to a subset of apicomplexans, which rely on gliding motility for cell invasion (**Figure 6**). These same patterns were confirmed by an independent phylogeny based on maximum likelihood (see supplemental **Figure S2**), indicating they are robust to different phylogenetic methods of analysis.

We chose *T. gondii* to test the importance of these two altered residues, since it is more amenable to genetic analyses. TgACTI residues were substituted with the corresponding amino acids from human muscle actin. The substituted proteins TgACTI-G200S (hydrogen bond substitution), TgACTI-K270M (hydrophobic loop substitution) and TgACTI-G200S/K270M (double substitution) were expressed using baculovirus and purified with Ni-affinity chromatography (**Figure 5C**). To determine if the substituted TgACTI alleles were more stable, purified proteins were incubated in F buffer and light scattering was used to examine polymerization. Wild type TgACTI underwent only limited polymerization while the TgACTI-K270M substituted protein showed a modest enhancement (**Figure 5D**). However, TgACTI-G200S and TgACTI-G200S/K270M showed increased polymerization, with both the rate (slope of the initial phase) and maximum extent being greater than wild type protein (**Figure 5D**).

**Figure 7:**
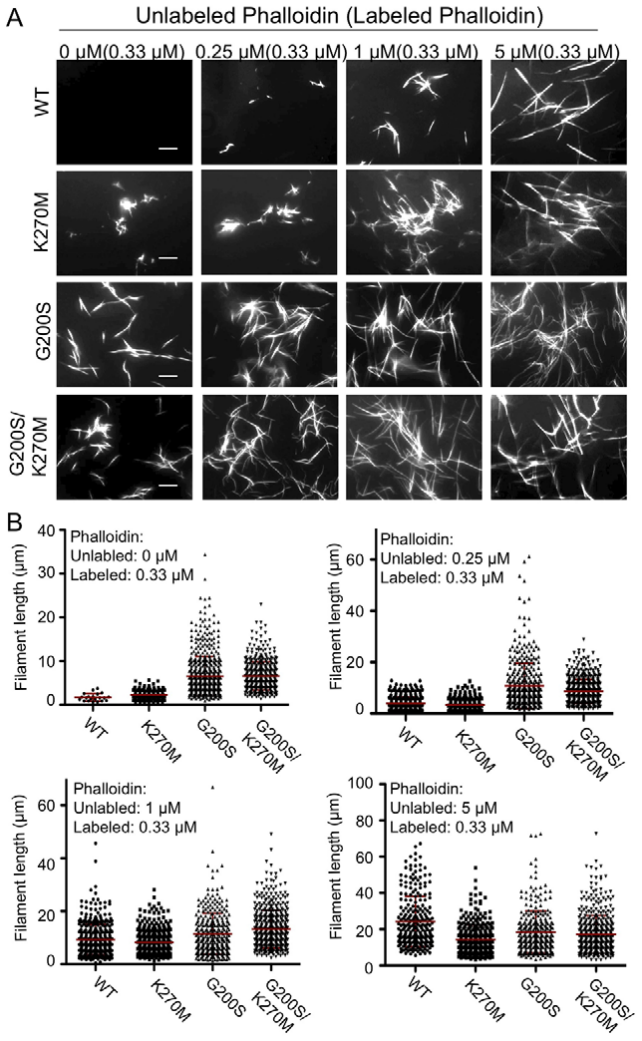
Substituted TgACTI alleles demonstrate enhanced *in vitro* polymerization. (A) *In vitro* polymerization of recombinant TgACTI alleles substituted with mammalian actin residues. Actins (5 µM) were visualized by fluorescence microscopy using 0.33 µM Alexa 488-phalloidin and different molar ratios of unlabeled phalloidin to actin. Scale bars, 5 µm. Representative of three or more similar experiments. The values at the top of the figure panel indicate the amounts of phalloidin used: µM unlabeled phalloidin (µM labeled phalloidin). (B) Quantitation of filaments formed during *in vitro* polymerization of substituted TgACTI alleles. Mean ± S.D. shown by horizontal line. Concentrations of phalloidin used for treatment are indicated below the graphs as µM unlabeled phalloidin (µM labeled phalloidin). In the presence of only low levels of labeled phalloidin (*i.e.* 0.33 µM), filaments formed by TgACTI-K270M (*P*<0.01), TgACTI-G200S (*P*<0.001), and TgACTI-G200S/K270M (*P*<0.001) were significantly longer that wild type TgACTI (Student's *t*-test).

The results of the light scattering assays were confirmed using fluorescent phalloidin staining and visualization via fluorescence microscopy. In all cases, filaments were visualized in the presence of low levels of labeled phalloidin (0.33 µM) combined with different amounts of unlabeled phalloidin. Wild type TgACTI (WT) required addition of 0.25 µM of unlabeled phalloidin to form small filaments and long filaments only formed upon addition of 5 µM (a 1∶1 ratio with actin) (**Figure 7A**, top row). The TgACTI-K270M protein showed a slight enhancement in polymerization with small filaments appearing in the presence of low levels of phalloidin and reaching longer lengths with addition of 1 µM molar ratio of unlabeled phalloidin (**Figure 7A**, second row). Interestingly, the TgACTI-G200S substitution showed much more robust polymerization with short filaments being detected even in the presence of low levels of labeled phalloidin (0.33 µM) and longer filaments appearing with addition of 0.25 µM of unlabeled phalloidin (**Figure 7A**, third row). TgACTI-G200S/K270M also formed longer filaments than seen with wild type TgACTI, similar to the TgACTI-G200S single mutant (**Figure 7A**, bottom row). Quantitation of the filament lengths formed by different TgACTI alleles confirmed that the features seen in microscopy were consistent across different replicate samples. All three of the mutant actins formed significantly longer filaments in low levels of phalloidin needed for visualization (*i.e.* 0.33 µM) compared to wild type TgACTI (**Figure 7B**). At higher levels of phalloidin, all of the actins formed long filaments of approximately equal length (**Figure 7B**). Taken together, these results show that TgACTI filaments may be inherently unstable due to the presence of only a few differences from conventional actins and that mutations designed to mimic mammalian actin in TgACTI result in formation of more stable actin filaments *in vitro*. We also investigated the effects of altering yeast actin to mimic residues found in TgACTI (*i.e.* converting S199 to G and L269 to K): neither mutation alone, nor the combination, showed dramatic change in filament assembly or length (data not shown), indicating there also must be other structural and kinetic differences that explain the extremely stable nature of actin filaments in yeast and likely other conventional actins. However, these data are not inconsistent with the gain of function results seen in mutant forms of TgACTI, where the magnitude of polymerization is still relatively modest when compared to yeast.

### Expression of stabilized TgACTI in *T. gondii*

**Figure 8:**
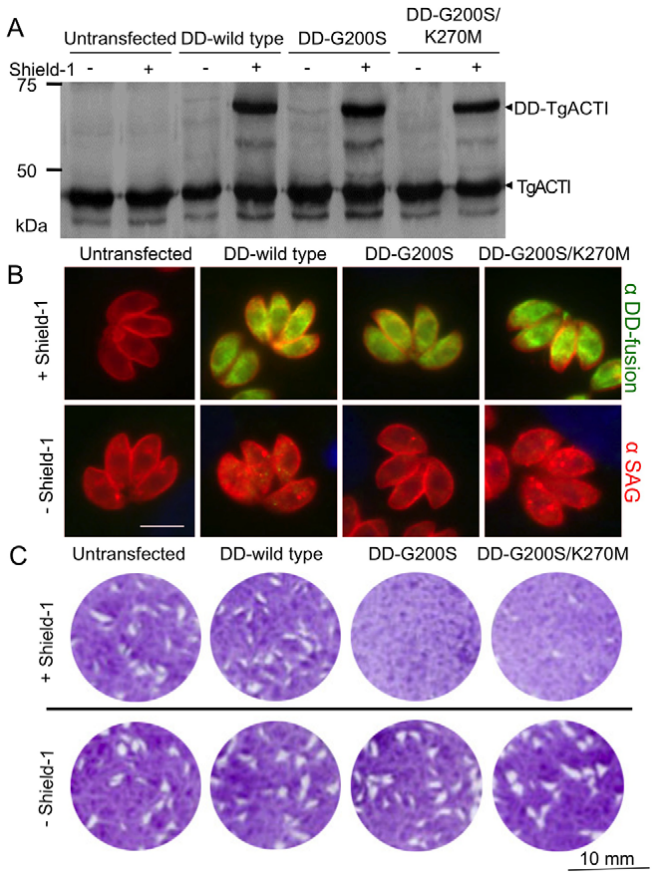
Expression of degradation domain (DD)-tagged TgACTI alleles in *Toxoplasma*. (A) Expression of DD-TgACTI fusion proteins following treatment ± Shield-1 for 40 hr and detected by Western blot with anti-TgACTI antibody. All strains express the endogenous TgACTI while the fusion proteins (DD-TgACTI) were only expressed by the transfected strains in the presence of Shield-1. (B) Expression of DD-tagged TgACTI alleles following treatment ± Shield-1 for 24 hr and stained for immunofluorescence with anti-SAG1 (surface antigen 1, red) and anti-c-myc (green) to detect the DD-TgACTI fusion protein. (C) Effects of DD-TgACTI allele expression on plaque formation. HFF monolayers were infected with untransfected parasites or those expressing DD-TgACTI alleles ± Shield-1 for 7 days and visualized by crystal violet staining. Representative of three or more similar experiments.

Despite the relatively small changes in actin stability observed in TgACTI mutants *in vitro*, we reasoned that such changes might still adversely affect actin-based processes in the parasite, such as gliding motility, which is exquisitely sensitive to actin stabilizing drugs like JAS [17]. To examine the effect of expressing stabilized mutants of TgACTI in *T. gondii*, we generated transgenic parasites expressing a second copy of TgACTI fused to an N-terminal degradation domain (DD), which allows regulated expression in the presence of Shield-1 [37]. This approach was chosen over allelic replacement, since we reasoned that the mutant alleles might be detrimental, hence compromising attempts to evaluate their functions. The regulated nature of the DD-stabilized proteins also allows the timing of expression to be controlled, thus minimizing the chance for pleomorphic downstream effects or compensatory changes that can occur using conventional dominant negative strategies. Transgenic lines expressing the DD-fusion proteins were tested for regulated expression by Western blot using an antibody against TgACTI (**Figure 8A**) and by immunofluorescence detection of the c-myc tag also present at the N-terminus (**Figure 8B**). The level of DD-tagged actins was approximately 50% of wild type actin and the patterns of staining was diffuse in the cytosol, similar to the pattern for endogenous actin described previously [13]. The expression of DD-tagged actins was similar at 6, 12, 24, and 40 hr (the point of natural egress) (see supplemental **Figure S3**). This relatively rapid induction with continued expression maintained over time allowed us to test different biological phenotypes at different time points. Initially, the impact of expressing DD-TgACTI fusions on the life cycle of the parasite was tested under continuous treatment with Shield-1 using a plaque assay, which monitors the normal intracellular growth cycle (**Figure 8C**). Although parasites expressing DD-G200S or DD-G200S/K270M formed plaques comparable to the controls in the absence of Shield-1, plaque formation was almost non-existent when parasites were treated with Shield-1 (**Figure 8C**), demonstrating that expression of stabilized TgACTI disrupts the parasite life cycle. In contrast, expression of the wild type DD-TgACTI (DD-wild type) had no effect on plaque formation either in the absence or presence of Shield-1 (**Figure 8C**). Additionally, growth in Shield-1 did not significantly alter cell division during the first 36 hr (**see supplementary Figure S3**), indicating that the expression of wild type or mutant actins does not affect endodyogeny, although we cannot rule out the possible effects on growth from expression of these actins over longer time frames.

**Figure 9:**
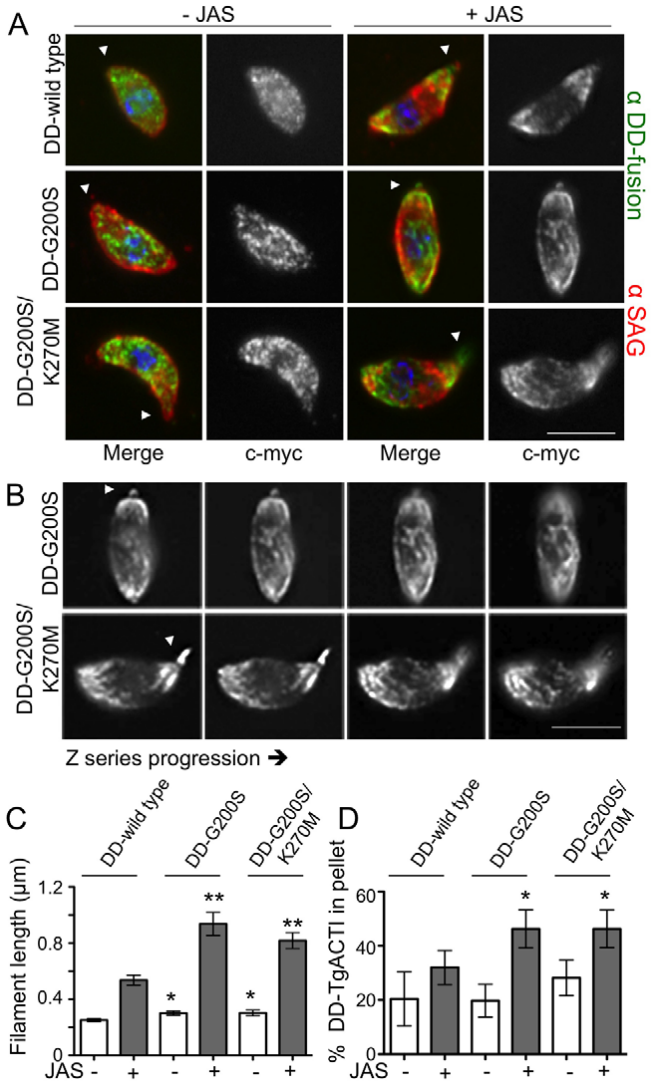
Stabilized actin alleles are more sensitive to JAS-stabilization than endogenous TgACTI in *Toxoplasma*. (A) Localization of c-myc-tagged DD-TgACTI alleles in parasites treated with Shield-1 for 40 hr as visualized by immunofluorescence with anti-c-myc antibody (green) and SAG1 (red). Treatment with low levels of JAS (*i.e.* 0.25 µM) induced spiral patterns of filaments in parasites expressing stabilized actin alleles (right). Apical end noted with arrowhead. Scale bar, 5 µm. (B) Images from (A) shown as z-slices (∼0.3 µm). Actin spirals in JAS-treated parasites were visualized by staining with anti-c-myc antibody. Apical end noted with arrowhead. Scale bar, 5 µm. (C) Quantitation of length of actin puncta and spirals formed in individual parasites treated with ±0.25 µM JAS. Mean ± S.D. * *P*<0.05 ** *P*<0.005 (Student's *t*-test) *vs.* DD-wild type with same JAS treatment. (D) Sedimentation analysis of F actin in parasites expressing DD-TgACTI fusions and treated ± Shield-1 for 40 hr. Cell lysates were prepared ±0.5 µM JAS, sedimented for 1 hr at 350,000*g* and analyzed by SDS-PAGE and quantitative Western blotting. Mean ± S.D., n = 3 experiments. * *P*<0.05 (Student's *t*-test) *vs.* DD-wild type.

Based on the observation that culturing parasites in Shield-1 for an entire lytic cycle does not affect replication, we treated cells for 40 hr and harvested parasites to examine the distribution and polymerization state of actin. Parasites expressing DD-TgACTI fusion proteins revealed a diffuse pattern of actin staining with some discrete puncta (**Figure 9A**; data not shown). The absence of detectable long filaments in cells expressing stabilized actins suggest that they behave somewhat differently *in vivo* than *in vitro*, perhaps as a result of other proteins that regulate actin turnover. Actin dynamics are highly sensitive to actin-stabilizing compounds like JAS, which permeates cells and stabilizes actin filaments [38]. Hence, we examined the distribution of actins in parasites expressing DD-TgACTI alleles following treatment with low levels of JAS (i.e. 0.25 µM). Filamentous actin structures were revealed emanating from both the apical and posterior poles in parasites expressing the stabilized TgACTI mutants grown in the presence of Shield-1, whereas staining of wild type DD-TgACTI relocalized to the poles without forming visible filaments (**Figure 9A**). The actin filaments seen in parasites expressing stabilized mutants of TgACTI formed spiral patterns beneath the surface of the parasite, as visualized in sequential slices of a z-series (**Figure 9B**). Measurement of the size of actin structures in parasites revealed that larger puncta were found in parasites expressing DD-G200S and DD-G200S/K270M *vs.* DD-wild type TgACTI in the absence of JAS, and that these mutant actins formed considerably longer filaments in the presence of low levels of JAS (**Figure 9C**). Co-staining of parasites expressing DD-tagged actins and treated with higher level of JAS (i.e. 1 µM), revealed that the tagged alleles co-localized in actin-filament rich apical projections, which are induced by JAS (see supplemental **Figure S4**).

To determine if the stabilized DD-TgACTI alleles polymerized more readily *in vivo*, we examined the proportion of globular and filamentous actin based on sedimentation at 350,000*g*, conditions previously found to be necessary to pellet short filaments that form in parasites [16]. Although no change in sedimented actin was detected in control lysates, treatment with low levels of JAS (i.e. 0.25 µM) induced much greater polymerization of the DD-G200S and DD-G200S/K270M mutants compared to DD-wild type (**Figure 9D**). Collectively, these studies demonstrate that stabilized forms of TgACTI were more sensitive to JAS-induced polymerization *in vivo*.

### Toxoplasma expressing stabilized actin undergo aberrant gliding motility

**Figure 10:**
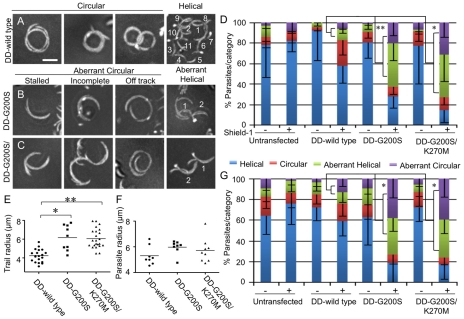
Parasites expressing stabilized actin undergo aberrant gliding motility. (A) Representative composite videos of normal gliding motility by parasites expressing DD-wild type TgACTI. (B,C) Representative composite videos of gliding by parasites expressing stabilized actin mutants revealed examples of stalled, incomplete or off-track circular patterns and disrupted helical patterns that were classified as aberrant. For helical gliding, the number of complete turns made during the time-lapse sequence are numbered. Images are composite frames from 60 sec of video recording. Scale bars, 5 µm. (D) Quantification of number of parasites undergoing each category of gliding motility following treatment ± Shield-1 for 40 hr. The percentage of parasites undergoing aberrant gliding (as defined in B,C) increased in the presence of Shield-1 in the mutant actins as compared to wild type, * *P*<0.05 ** *P*<0.01, (Student's *t*-test). Mean ± S.D. (E) Comparison of radii of circular tracks formed during normal gliding by DD- wild type expressing parasites *vs.* parasites expressing mutant actins that formed aberrant circular tracks (*i.e.* stalled or off-track). * *P*<0.001 ** *P*<0.0001 (Mann-Whitney test). Mean shown by horizontal line. (F) Comparison of parasite curvature during normal gliding by DD-wild type expressing parasites *vs.* parasites expressing mutant actins that formed aberrant circular tracks (*i.e.* stalled or off-track). Data shown are the average curvature radii of individual parasites during gliding motility. (G) Quantification of number of parasites undergoing each category of gliding motility following treatment ± Shield-1 for 6 hr. The percentage of parasites undergoing aberrant gliding (as defined in B, C) increased in the presence of Shield-1 in the mutant actins as compared to wild type, * *P*<0.001, (Student's *t*-test). Mean ± S.D.

To examine the impact of stabilized TgACTI alleles on parasite motility, we employed video microscopy to analyze the typical circular and helical motions that are characteristic of gliding, as described previously [7]. In contrast to DD-wild type TgACTI expressing parasites that underwent normal circular gliding (**Figure 10A**, see supplemental **Video S1**), a large percentage of the circular movements in parasites expressing DD-G200S and DD-G200S/K270M actins were aberrant (**Figures 10B, 10C**). For example, DD-G200S and DD-G200S/K270M expressing parasites often stalled, were unable to complete circles, or went off-track during gliding (**Figures 10B, 10C** see supplemental **Videos S3, S4, S5**). Quantification of these results indicated that expression of the DD-wild type allele resulted in a higher frequency of circular gliding than helical, relative to untransfected parasites, however these movements were largely normal (**Figure 10D**). Although mutants expressing DD-G200S and DD-G200S/K270M underwent wild type motility in the absence of Shield-1, significantly more cells exhibited aberrant forms of gliding motility in the presence of Shield-1 (**Figure 10D**). Comparison of the radii of tracks made by DD-wild type and DD-G200S and DD-G200S/K270M expressing parasites undergoing circular gliding motility revealed that the mutants traced out partial arcs that were significantly larger than circular tracks formed by wild type parasites (**Figure 10E**). The larger arc traced by parasites expressing stabilized actins was in part due to a modest change in the shape of the cell, as shown by measuring the curvature of the parasite body during gliding, although this difference was not significant (**Figure 10F**). Collectively, these results indicate that expression of the mutant DD-G200S and DD-G200S/K270M forms of TgACTI disrupts normal circular gliding motility.

Consistent with previous descriptions of normal helical motility [7], DD-wild type expressing parasites underwent helical gliding at a relatively fast rate and moved through numerous corkscrew motions, noted in the example shown (**Figure 10A** see supplemental **Video S2**). In contrast, DD-G200S and DD-G200S/K270M expressing parasites were delayed in their movements and went through fewer flips and turns (**Figures 10B, 10C**, see supplemental **Video S6**). Parasites expressing the stabilized TgACTI alleles were significantly slower in both helical and circular gliding compared to the untransfected or DD-wild type parasites (**Table 1**). Taken together, these findings reveal that expression of stabilized mutants of TgACTI significantly disrupts gliding motility in *T. gondii*.

**Table 1:** Quantification of *T. gondii* gliding motility rates from video microscopy.

Strain	Shield-1	Normal Helical^a^	Aberrant Helical	Normal Circular	Aberrant Circular
Untransfected	**−**	1.34±0.23^b^	**-** c	**-**	-
Untransfected	**+** d	1.50±0.44	**-**	**-**	-
DD-wild type	**−**	1.54±0.29	**-**	**-**	-
DD-wild type	**+**	1.35±0.28	**-**	1.09±0.40	-
DD-G200S	**−**	1.49±0.32	**-**	1.35±0.46	-
DD-G200S	**+**	**-**	0.72±0.32***	**-**	0.71±0.35*
DD-G200S/K270M	**−**	1.59±0.41	**-**	1.32±0.26	**-**
DD-G200S/K270M	**+**	**-**	0.73±0.22***	**-**	0.64±0.28**

a µm/sec.

b Mean ± S.D.

c Too infrequent for analysis.

d 4 µM Shield-1.

*: Comparison of circular gliding by DD-G200S ± Shield-1, *P*<0.05, Student's *t*-test.

**: Comparison of circular gliding by DD-G200S/K270M ± Shield-1, *P*<0.01, Student's *t*-test.

***: Comparison of helical gliding by DD-G200S or DD-G200S/K270M ± Shield-1, *P*<0.005, Student's *t*-test.

Since earlier data had shown that DD-tagged alleles are induced to similar levels after 6 hr of treatment, we wanted to test the effects of shorter treatments on gliding motility, in order to rule out possible indirect effects of long-term treatment. Hence, Shield-1 was added to infected monolayers during only the last 6 hr of development, and parasites were harvested following natural egress. As described above, defects in gliding motility were seen in *T. gondii* parasites expressing either the DD-G200S and DDG200S/K270M mutant actins (**Figure 10G**). Following Shield-1 treatment, parasites expressing mutant, but not wild type actin, showed increased frequency of aberrant circular trails including stalled, incomplete and off-track patterns, as well as aberrant helical patterns (**Figure 10G**). The similar phenotypes observed at 6 hr and 40 hr of Shield-1 treatment for the mutant actins, and absence of defects in parasites expressing wild type tagged actin, indicates that the aberrant gliding phenotypes are due to expression of stabilized actin alleles, rather than non-specific effects.

## Discussion

Our studies suggest that the instability of parasite actin filaments is an inherent property that results in part from differences in monomer-monomer interactions that normally stabilize the filament. Although parasite actins only form clusters of small filaments when visualized with low levels of phalloidin, equimolar levels of phalloidin rescued parasite actin filament instability *in vitro*, resulting in long stable filaments that resembled conventional actin. Reversion of two key residues in *T. gondii* actin to match those predicted to stabilize mammalian muscle actin, also partially restored filament stability *in vitro*. Furthermore, *in vivo* expression of these stabilized actins led to disruption of gliding in *T. gondii*. These findings provide insight into the molecular basis of parasite actin filament dynamics and reveal formation of short, highly dynamic actin filaments is an important adaptation for parasite motility.

Our studies are in agreement with previous work on the polymerization properties of parasite actins and extend these findings by examining the molecular basis for instability of actin filaments. We have previously reported that TgACTI undergoes polymerization *in vitro* as determined by tryptophan quenching and sedimentation, although the extent of this process was not compared to conventional actins [23]. In the present report, we examined actin polymerization by staining with fluorescently labeled phalloidin, sedimentation, electron microscopy, and light scattering, which provides a convenient method to study dynamics. Our findings indicate that while TgACTI undergoes polymerization, it has a very limited capacity to do so in comparison to yeast actin. There have been previous studies on the polymerization differences between muscle actin and actins from either budding [39] or fission yeast [40], however the differences observed in these cases are relatively minor compared to what we observe here between parasite and yeast actins. Rather than subtle shifts in the polymerization kinetics or critical concentration, the extent of polymerization with these parasite actins is fundamentally different from yeast or mammalian actins.

The inefficient polymerization of TgACTI was rescued by conditions that stabilize the filament including treatment with phalloidin *in vitro*. Other studies have previously examined PfACTI produced in yeast and concluded that it also polymerizes poorly *in vitro* [19]. In this prior study, stabilization with phalloidin (1∶4 molar ratio) was used to achieve modest levels of polymerization of PfACTI purified from yeast and copolymerized with bovine ß-actin [19]. In a separate study, the ability of PfACTI purified from merozoites to be stabilized by phalloidin showed a pH dependence, with greater polymerization detected at pH 6.0 than 8.0 [24], although the basis of this response is unknown. Our findings with PfACTI and PfACTII demonstrate that these actins, while modestly better at polymerization than TgACTI, also fail to polymerize robustly on their own. The reasons for this apparent instability have not been definitively resolved but could result from a lower capacity for elongation, more rapid disassembly, or a lower capacity to anneal, as described previously [41]. In contrast to yeast and vertebrate actins, our studies show that apicomplexan actins are highly dependent on addition of high levels (*i.e.* equimolar ratios) of phalloidin to form long stable filaments. Because the phalloidin binding site sits at the interface between protomers within the filament, it may overcome inherent instability caused by changes that affect monomer-monomer contacts within parasite actin filaments.

Several new F-actin models have been produced in the past few years [32], [42] and these models have given us new insight into the structural details of protomer interactions within the filament. However, the difficulties with interpreting a single, uniform F-actin structure have also been highlighted [43], and this is precisely why we make use of molecular modeling studies in our work here. Based on our dynamics simulations, we see that actin filaments are stabilized by interactions across the width (inter-strand) of the filament through two key regions including the “hydrophobic plug” encompassing residues 265–270 and a helix from residues 191–199 [32], [42]. Our studies further suggest that relatively few changes in these critical regions account for the instability of parasite actin filaments. Among these alterations, a change in the hydrophobic plug (*i.e.* K270 in *T. gondii*) plays a modest role while an alteration in the helix (*i.e.* G200) has a larger affect on filament stability. The substitution of K270M in TgACTI resulted in filaments that were detected by fluorescent staining at low concentrations of phalloidin, although this change had less effect on actin polymerization as monitored by light scattering assays in the absence of phalloidin. As this residue lies within the phalloidin pocket, it suggests that hydrophobic residues here result in enhanced phalloidin binding. Mutations designed to reduce hydrophobicity in the corresponding residue in yeast actin (*i.e.* L269) have no affect on polymerization, while those at the other end of the hydrophobic plug are much more severe [44]. Hence, these results indicate that K270 contributes to normally low phalloidin binding of parasite actins, while it likely plays a lesser role in intrinsic filament instability. Modeling predictions also indicate that S199 in muscle actin plays a role in filament stabilization via a hydrogen bond network with R177 and D179. Consistent with this, mutation of G200S had a larger impact on the *in vitro* polymerization of TgACTI as shown by increased light scattering, even in the absence of phalloidin. Collectively, the absence of these two stabilizing interactions in TgACTI partially explains the inherent instability of parasite actin filaments. Intriguingly, both PfACTI and PfACTII polymerized slightly better than TgACTI in the absence of stabilizing agents, and the introduction of two alterations in TgACTI (G200S and K270M) resulted in polymerization to levels that approximated with wild type levels of the *Plasmodium* actins (compare Figures 1,2 to [Fig ppat-1002280-g003] [Fig ppat-1002280-g004] 5,7). Together, these findings indicate that other sequence and structural differences between these actins must contribute to their inherent differences in polymerization kinetics, which is not surprising in light of findings that even conventional actins such as yeast and muscle differ significantly [39], [40].

The intrinsic properties of actins may be highly significant in controlling dynamics in apicomplexan parasites since they contain only a streamlined set of actin-binding proteins [20], [21]. PfACTI contains similar substitutions to those described for TgACTI above, while PfACTII contains a K at 270 and T at 200 instead of S199 in muscle. PfACTI is expressed throughout the *Plasmodium* life cycle including merozoites, while PfACTII is expressed primarily in sexual stages ([14], [15] and EupathDB.org). Sporozoites and ookinetes undergo actin-dependent gliding motility on substrates and cells, while merozoites do not show substrate-dependent gliding but rely on a similar actin-dependent process for invading red blood cells [45]. In comparing the two different actin isoforms in *Plasmodium*, PfACTII was slightly more stable than PfACTI as shown by fluorescent phalloidin staining of filaments, raising the possibility that T200 is capable of partial hydrogen bonding, analogous to the interaction of S199 in muscle actin. Increased actin filament stability may be important in non-motile forms such as gametocytes where PfACTII is highly expressed [15]. It is also possible that the natural variation in actins found in parasite actins, and the specific changes in TgACTI mutants studied here, are influenced by interactions with actin binding proteins.

Actin filament instability is evidently an important adaptation since expression of stabilized TgACTI within the parasite had a detrimental effect on gliding motility, while only modestly affecting cell division over the first 24–36 hr. Although we have not directly measured the effects on invasion or egress, these processes also depend on gliding motility and therefore are likely affected by expression of the stabilized mutants of TgACTI. Collectively, these phenotypes likely have an additive effect in the plaque assay, which captures successive rounds of invasion, replication, egress, and motility, thus leading to a more dramatic phenotype. The effects of expressing mutant actins in *T. gondii* partially mimic the effects of treatment with JAS, supporting the conclusion that they arise by stabilizing actin filaments. Previous studies using actin stabilizing agents such as JAS have revealed that increased polymerization of TgACTI filaments adversely effects motility and host cell invasion [17], [26], [30]. In the present study, stabilized mutants of TgACTI were more sensitive than wild type parasites to JAS, as shown by formation of spiral actin filaments and increased sedimentation. The spiral patterns seen here are similar to those reported previously from wild type *T. gondii* treated with high levels of JAS [17]; however, notably here they occur with low levels of JAS and are only seen in mutants expressing stabilized TgACTI forms. Stabilized DD-TgACTI mutants also had a profound effect on disrupting normal motility in the absence of treatment, revealing that this phenotype is not simply due to enhanced binding to JAS or phalloidin. Intriguingly, parasites expressing stabilized actins formed circles with larger radii, moved more slowly, and stalled in the process of gliding. These larger arcs were in part due to a more relaxed curvature of parasites expressing mutant actins, although this difference was much less pronounced than that seen in the trails. Hence, the increased trail radii likely results from the parasite slipping off its track as it migrates around the circle. Previous studies have also shown that the degree of actin polymerization can influence adhesive strength and hence the gliding behavior of *Plasmodium* sporozoites [46]. Collectively these data suggest that short, highly dynamic actin filaments are required for parasites to complete the tight arcs and corkscrew turns that are characteristic for circular and helical gliding [47].

The current model for gliding motility predicts that short, highly dynamic actin filaments attached to transmembrane adhesive proteins are translocated along the surface of the parasite by a small myosin [48]. The myosin motor, which is anchored in the inner membrane complex [49], is also highly nonprocessive [50], meaning it does not stay attached to a single filament for long periods. Instead, this model predicts that short actin filaments, tethered to transmembrane adhesins, are passed sequentially between motor complexes that operate independently. Consistent with this, where actin filaments have been seen in parasites, they are quite short (*i.e.* 50–100 nm) [16], [23]. Actin in apicomplexans may be adapted for rapid turnover of short filaments, since long filaments would increase the likelihood of multiple motors being engaged simultaneously, potentially leading to conflicting forces on the same filament. Although we were not able to discern distinct filaments in parasites expressing DD-TgACTI proteins, the observed punctate staining pattern may reflect clusters of short filaments that are below the resolving power of the light microscope (in theory ∼200 nm, but in practice likely ∼400 nm). Nonetheless, we would predict based on their *in vitro* properties that the G200S and G200S/K270M mutants would form more stable filaments, which could inhibit motility by reducing free monomers needed for new filament assembly, or by physically disrupting productive motor-actin filament complexes. Alternatively, stabilized DD-TgACTI mutants could affect interactions with actin-binding proteins *in vivo*, including those involved in polymerization or depolymerization. Although apicomplexans lack an Arp2/3 complex [22], they express several formins that act to increase actin polymerization [51], [52] and actin depolymerization factor, which acts primarily to sequester monomers and prevent polymerization [53]. Regardless of the exact mechanism, our results indicate that even subtle changes in actin filament stability significantly affect function, underscoring the importance of rapid actin dynamics in apicomplexans.

In comparing apicomplexans to other organisms, the G200S mutation is found in a subset of apicomplexans including *Toxoplasma*, *Neospora*, *Eimeria*, and *Plasmodium* spp. but excluding *Theileria*, *Babesia*, *Cryptosporidium*, and gregarines. Hence it is uncertain if conversion to G200 arose in the common ancestor of coccidians (monoxenous and tissue cyst forming) and hematozoa and was subsequently lost by some members, or if arose independently in *Plasmodium* and the coccidian. Gliding motility has not been described in *Thieleria*, which enters lymphocytes by a very different process than other apicomplexans [54]. However, *Babesia* enters red cells by a process very analogous to that seen in *Plasmodium* [55], and so likely has a conserved mechanism for actin-based motility. *Cryptosporidium* and gregarines also move by gliding motility [6], although the polymerization properties of actins from these organisms have not been examined. Hence it is unclear whether these other apicomplexans rely on more stable actins, or if other divergent residues impart similar properties to those observed in *Toxoplasma* and *Plasmodium*.

The substitution of K/R in the hydrophobic plug at residue 269/270 is seen in a wider variety of protozoa including dinoflagelates, ciliates, and apicomplexans. Consistent with this, diverse actins from protozoans *Leishmania* [56], *Giardia* [57], and *Tetrahymena* [58] have also been reported not to bind well to phalloidin and to display unusual polymerization kinetics or novel actin structures. This pattern further suggests that stable actin filaments are a more recent evolutionary development, found in amoeba, yeast, plants and animals, but not shared by many protozoans. There are some exception to this pattern, such as *Giardia*, which expresses a very divergent actin that nonetheless forms stable filamentous structures [57]. Although no kinetic measurements have been reported for *Giardia* actin as of yet, when available they will provide extremely useful comparisons to other systems. Overall these differences in actin filament stability likely reflect adaptations for stable *vs.* dynamic actin cytoskeletons that are designed for very different life strategies. The importance of dynamic actin turnover in apicomplexans is shown by introduction of stabilizing residues in TgACTI, changes that were sufficient to dramatically slow the speed of gliding and result in aberrant forms of motility. Collectively, these findings demonstrate that actin filament instability and rapid turnover are important adaptations for productive gliding in apicomplexans, and suggest that small molecules designed to selectively stabilize parasite actins may have potential for preventing infection.

## Materials and Methods

### Plasmid constructions and transfection

Recombinant *Toxoplasma*, *Plasmodium* and yeast actins were expressed in baculovirus, as previously described [23]. Recombinant viruses were created by amplification from 3D7 strain of *Plasmodium falciparum* cDNA or *Saccharomyces cerevisiae* cDNA using gene-specific primers (Table S1) and the resulting products were cloned into the viral transfer vector pAcHLT-C (BD Biosciences Pharmingen). Recombinant viruses were obtained by cotransfection with linearized baculogold genomic DNA into Sf9 insect cells (BD Biosciences Pharmingen), according to manufacturer's instructions. Recombinant viruses for mutant TgACTI alleles and were created via site-directed mutagenesis using wild type TgACTI as a template and allele-specific primers (Table S1).

### Actin expression and purification

Hi5 insect cells were maintained as suspension cultures in Express-Five SFM media (Invitrogen). Hi5 cells were harvested at 2.5 days postinfection with recombinant virus and lysed in BD BaculoGold Insect Cell Lysis Buffer (BD Biosciences Pharmingen) supplemented with 0.2 mM CaCl_2_, 0.2 mM ATP, 0.2 mM NaN_3_, and protease inhibitor cocktail (E64, 1 µg ml^−1^ AEBSB, 10 µg ml^−1^; TLCK, 10 µg ml^−1^; leupeptin, 1 µg ml^−1^). His-tagged actins were purified using Ni-NTA agarose (Invitrogen). After binding for 2 hr, the column was washed sequentially with G actin buffer without DTT (G-DTT buffer) (5 mM Tris-Cl, pH 8.0, 0.2 mM CaCl_2_, 0.2 mM ATP), then G-DTT buffer with 10 mM imidazole, G-DTT buffer with 0.5 M NaCl and 10 mM imidazole, G-DTT buffer with 0.5 M KCl and 10 mM imidazole, and finally G-DTT buffer with 25 mM imidazole. Proteins were eluted with serial washes of G-DTT buffer containing 50 mM, 100 mM, and 200 mM imidazole, pooled together and dialyzed overnight in G-actin buffer containing 0.5 mM DTT with 100 µM sucrose. Purified recombinant actins were clarified by centrifugation at 100,000*g*, 4°C, for 30 min using a TL100 rotor and a Beckman Optima TL ultracentrifuge (Becton Coulter) to remove aggregates. Purified proteins were resolved on 12% SDS-PAGE gels followed by SYPRO Ruby (Molecular Probes) staining, visualized using a FLA-5000 phosphorimager (Fuji Film Medical Systems), and quantified using Image Gauge v4.23. Purified actins were stored at 4°C and used within 2–3 days.

### Fluorescence microscopy of actin filaments

Purified recombinant actins were clarified as described above and incubated (5 µM) in F buffer (50 mM KCl, 2 mM MgCl_2_, 1 mM ATP), and treated with different molar ratios of unlabeled phalloidin to actin from 0∶1 to 1∶1 (Molecular Probes). In addition, final concentrations of 0.13 µM or 0.33 µM Alexa-488 phalloidin (Molecular Probes) were added to each sample to visualize filaments. Following polymerization for 1 hr, samples were placed on a slide and viewed with a Zeiss Axioskop (Carl Zeiss) microscope using 63× Plan-NeoFluar oil immersion lens (1.30 NA). Images were collected using a Zeiss Axiocam with Axiovision v3.1 and processed using linear adjustments in Adobe Photoshop v8.0. Filament lengths were determined using the measurement feature of Axiovision software (Zeiss). For each actin sample, filaments were measured from 8–10 fields (63×) within three biological replicates.

### Light scattering

Purified recombinant actins were clarified as described above and incubated (5 µM) in G buffer containing 1 mM EGTA and 50 µM MgCl_2_ for 10 min (to replace bound Ca^2+^ with Mg^2+^). Samples were placed in a submicrocuvette (Starna Cells) and following addition of 1/10^th^ volume of 10× F buffer, light scattering was monitored with the PTI Quantmaster spectrofluorometer (Photon Technology International) with excitation 310 nm (1 nm bandpass) and emission 310 nm (1 nm bandpass). Curves were processed by second order smoothing with 15–30 neighbors using Prism (Graph Pad).

### Parasite actin homology models and alignment

Homology models for TgACTI, PfACTI, and PfACTII sequences were built on the ADP-actin crystal structure (1J6Z) [59] using Modeller [60]. Homology models were aligned and visualized using VMD [61]. Protein sequences for actins from *Homo sapiens* (muscle α-actin), gi: 6049633; *Saccharomyces cerevisiae*, gi: 38372623; *Toxoplasma gondii*, gi: 606857; *Plasmodium falciparum* ACTI, gi: 160053; and *Plasmodium falciparum* ACT2, gi: 160057; were aligned using DNASTAR Lasergene MegAlign v7 and modified using Adobe Illustrator v10.

### Actin structure for molecular modeling

An atomic model of phalloidin was derived from the solid state structure of a synthetic derivative [62], modified to contain dihydroxy-Leu7 using Maestro (Schrödinger LLC,) and energy minimized using MacroModel (Schrödinger LLC,) with a MMFF94s forcefield. The model was further optimized in continuum solvent using Jaguar (Schrödinger LLC), with DFT level of theory using a hybrid B3LYP functional and 6-31G** basis set. The actin filament model based on X-ray fiber diffraction data [32] was used to create an 8-monomer filament of muscle F-actin. A 50 ns molecular dynamics (MD) simulation in explicit water was carried out using NAMD [63] in an NpT ensemble with a pressure of 1 atm and a temperature of 300 K with explicit TIP3P water. CHARMM27 forcefield was used with a 10 Å cut off for van der Waals with a 8.5 Å switching distance, and Particle Mesh Ewald for long-range electrostatics. Bonded hydrogens were kept rigid to allow 2 fs time steps. A simulated annealed structure of muscle filament model with phalloidin in the binding site was used as the template for building parasitic actin filament homology models using Modeller [60].

### Docking studies

Docking of phalloidin to different sites along the filament was captured using multiple snapshots taken at intervals of 200 ps from the 50 ns simulation. AutoDock [64] was used to perform large scale docking runs with a coarse grid that covered the six binding sites on the filament. To determine the correct orientation of phalloidin in the binding site, higher resolution docking studies were performed on each binding site using both AutoDock and Glide (Schrödinger LLC) in independent trials and clustered to derive the most probable docking orientation. For AutoDock, flexible ligand docking was performed using Lamarckian genetic algorithm with a population size of 200, 10 million energy evaluations, and a local search probability frequency at 0.2. Grid spacings of 0.325 Å and 0.25 Å were used for coarse and high resolution docking, respectively, and the results were clustered at RMSD of 3.0 Å from the lowest docked energy conformer. Gasteiger-Marsili charges were assigned to the ligand using Sybyl (Tripos Inc.,). Default parameters were used for Glide; ligand charges were derived from the quantum optimization calculation and protein charges were derived from the OPLS2001 forcefield.

### Conditional expression system

TgACTI alleles were amplified by PCR and inserted into a modified vector pTUB-DD-myc-YFP-CAT-Pst1 [37] at unique Pst1-AvrII sites to generate DD-TgACTI fusions. The resulting plasmids were transfected into tachyzoites of the RH strain of *Toxoplasma* and parasites were single celled cloned on monolayers of HFF cells and propagated as previously described [65].

### Immunofluorescence microscopy

For intracellular staining, parasites were allowed to invade HFF monolayers on glass coverslips for 24 hr in the presence or absence of 4 µM Shield-1. The coverslips were then fixed with 4% formaldehyde and stained with mouse anti-c-myc (Zymed) to detect the DD-fusion proteins followed by goat anti-mouse IgG conjugated to AlexaFluor 488 (Molecular Probes) and mAb DG52 (anti-TgSAG1) directly conjugated to AlexaFluor 594 to detect the parasite. To examine the pattern of actin following expression of DD-tagged actins, parasites were cultured in Shield-1 for one lytic cycle (i.e. 40 hr) and then harvested following natural egress. Freshly harvested parasites were treated ±0.25 µM JAS (Invitrogen) for 15 min and allowed to glide for 15 min on glass coverslips coated with 50 µg ml^−1^ BSA. Coverslips were fixed and stained with mouse anti-cmyc (Zymed) followed by goat anti-mouse IgG conjugated to AlexaFluor 488 and mAb DG52 labeled with AlexaFluor 594. Coverslips were mounted in Pro-Long Gold anti-fade reagent (Invitrogen) and viewed with a Zeiss Axioskop (Carl Zeiss) microscope using 63× Plan-NeoFluar oil immersion lens (1.30 NA). Images were collected using a Zeiss Axiocam and deconvolved using a nearest neighbor algorithm in Axiovision v3.1. Images were processed using linear adjustments in Adobe Photoshop v8.0. To determine the length of actin filament structures, the longest continuously staining patterns (*i.e.* puncta, filaments, or spirals) were determined using the measurement feature of Axiovision software (Zeiss). Measurements were made from 6–8 separate parasites from each of the groups (*i.e.* DD-wild type, DD-G200S, and DD-G200S/K270M) ± JAS treatment.

### Plaque assay

Plaque assays were conducted by adding 300 purified parasites to HFF monolayers in 6-well dishes containing medium+DMSO or medium +3 µM Shield-1 in DMSO and incubated at 37°C with 5% CO_2_ for 7 days. Plates were then fixed with 70% ethanol and stained with 0.01% crystal violet to visualize plaques.

### Shield time course

Freshly lysed parasites were used to infect HFF monolayers with the addition of 4 µM Shield-1 for 6, 12, 24 or 40 hr prior to egress. Following natural egress, parasites were filtered, spun at 400*g* for 10 min and resuspended in Laemmli sample buffer. Parasite lysates from each time point were resolved on 12% SDS-PAGE gels, Western blotted with anti-TgACTI antibody, visualized using a FLA-5000 phosphorimager (Fuji Film Medical Systems) and quantified using Image Gauge v4.23.

### Replication assays

Analysis of replication of parasites expressing DD-TgACTI alleles were conducted by adding freshly egressed parasites to HFF monolayers on coverslips in 24 well plates containing medium +DMSO or medium+4 µM Shield-1 in DMSO and incubated at 37°C with 5% CO_2_ for 24 or 36 hr at which time the coverslips were fixed and stained with mAb DG52 labeled with AlexaFluor 488. Coverslips were mounted in Pro-Long Gold anti-fade reagent (Invitrogen) and viewed with a Zeiss Axioskop (Carl Zeiss) microscope using 63× Plan-NeoFluar oil immersion lens (1.30 NA). The numbers of parasites per vacuole were counted in triplicate from at least 50 vacuoles per coverslip from three replicate experiments.

### Actin sedimentation analysis

Parasite strains expressing DD-tagged actins were treated ±0.5 µM JAS for 30 min, lysed with Triton-X-100 for 1 hr, centrifuged at 1,000*g*, 4°C for 2 min and supernatants centrifuged at 350,000*g*, 4°C for 1 hr using a TL100 rotor and a Beckman Optima TL ultracentrifuge (Becton Coulter). Supernatant proteins were acetone precipitated and washed with 70% ethanol. All pellets were resuspended in 1× sample buffer, resolved on 12% SDS-PAGE gels, Western blotted with anti-TgACTI antibody, visualized using a FLA-5000 phosphorimager (Fuji Film Medical Systems), and quantified using Image Gauge v4.23.

### Video microscopy

Parasite gliding was monitored by video microscopy as previously described [7]. Parasites were treated with DMSO or 4 µM Shield-1 for 6 or 40 hr, resuspended in Ringer's solution and allowed to glide on uncoated glass coverslips. Images were captured with 50–100 ms exposure times at 1 sec intervals, combined into composites with Openlab v4.1 (Improvision), analyzed using ImageJ and saved as QuickTime videos. Cell motility was tracked using the ParticleTracker plug-in to evaluate average speeds from a 3–15 tracks. The percentage of parasites undergoing different forms of motility was quantified from 4 or more separate videos, 60 sec in length and containing 10–40 motile parasites each, using Cell Counter, as described [66]. Radii of circular trail patterns and of the curvature of gliding parasites were determined using the measurement feature of Axiovision software (Zeiss). Measurements of the radii of trails were made from tracks of individual parasites from 4 separate videos containing 10–40 motile parasites each. The curvatures of individual parasites were determined from parasites undergoing circular (DD-wild type) *vs.* off-track and stalled (DD-G200S, G200S/K270M) patterns of motility. The curvature of individual parasites was measured independently from 3–5 separate frames from a single motility track taken from representative time-lapse recordings.

### Phylogenetic analysis

Sequences of actins for 83 organisms including a variety of protists, plants, fungi and animals, were obtained from GenBank and aligned using Clustal [67] with a gap opening penalty of 30 and extension penalty of 0.75. The alignment (see supplementary **Figure 5**) was imported as a NEXUS file in the PAUP* [68] and used to generate tress by Neighbor-Joining distance using BioNJ and 1000 bootstrap replicates. Only branches of >50% were retained in building the consensus tree. Unrooted trees were drawn in TreeView [69]. Separately, the alignment file was imported as a nexus file into HyPhy [70] and used to generate a maximum likelihood tree under the HKY85 model with 100 bootstrap replicates.

### Statistical analysis

Statistics were calculated in Excel or Prism (Graph Pad) using unpaired, two-tailed Student's *t*-tests for normally distributed data with equal variances, and two-tailed Mann-Whitney analysis for analysis of samples with small samples sizes of unknown distribution. Significant differences were defined as *P*≤0.05.

## Supporting Information

Figure S1 Sequence alignment for comparison of actins from *Homo sapiens* (muscle) (Human), *Saccharomyces cerevisiae (Yeast)*, *Toxoplasma gondii (TgACTI)*, *Plasmodium falciparum* (PfACTI or PfACTII). Residues that were mapped to within 4 Å of the phalloidin-binding site in muscle actin are highlighted. Color code: Blue - positive charged residues (including His), Red - negative charged, Green – hydrophobic, Purple - polar residues, Orange - aromatic.
(TIF)

Figure S2 Maximum likelihood tree of diverse actins from a range of protists, fungi, plants and animals. The nexus output from the alignment file was the alignment file was imported into HyPhy [69] and used to generate a maximum likelihood tree under the HKY85 model with 100 bootstrap replicates. Red lines indicate taxa with K or R residues at 270, while green lines indicate taxa with G200 and K/R 270 (numbering based on *T. gondii*). See supplemental materials (**Figure S5**) for the alignment.
(TIF)

Figure S3 Time course of Shield treatment. A) Expression of DD-TgACTI fusion proteins following treatment ± Shield1 incubation for variable amounts of time (0, 6, 12, 24, 40 hr). All strains express the endogenous TgACTI while the fusion proteins (DD-wild type, DD-G200S or DD-G200S/K270M) were only expressed by the transfected strains in the presence of Shield-1. B) Replication of parasite strains expressing DD-TgACTI fusion proteins ± Shield1 at 24 and 36 hr. The numbers of parasites per vacuole were counted at the two time points. Mean ± S.D., n = 3 experiments.
(TIF)

Figure S4 DD-TgACTI localization in JAS-induced actin protrusions. Expression of DD-tagged TgACTI alleles following treatment ± Shield-1 for 40 hr and JAS treatment (1 µM) for 15 min. Parasites were stained for immunofluorescence with anti-TgACTI (red) and anti-c-myc (green) to detect the DD-TgACTI fusion protein and compare localization in the JAS-induced actin protrusions. Scale bar, 5 µm. Arrowheads depict the JAS-induced actin protrusions.
(TIF)

Figure S5 Clustal alignment of actins from a diverse set of protists, fungi, plants, and animals. Maximum likelihood tree was drawn using HyPhy (69) under the HKY85 model with 100 bootstrap replicates. The likelihood for the tree shown is Ln = −12,231. Branches are colored to indicate actins that contain substitutions. Red bars include organisms with K or R at residue 270 (yeast), which is typically hydrophobic and most often L or M in conventional actins (black bars). Green bars indicate organisms that have G at residue 200 (yeast), where most organisms have S, as well as the K270 substitution.
(PDF)

Figure S6 Enlarged parasite actin homology models. (A) Model of TgACTI (blue) mapped onto PfACTI (red) highlighting amino acid differences (yellow). (B) Model of PfACTI (red) mapped onto PfACTII (green) highlighting amino acid differences (yellow).
(TIF)

Table S1 Primer list for construction of vectors used in current study.
(DOCX)

Video S1 DD-wild type parasites undergo normal circular gliding. DD-wildtype parasites were treated with Shield-1 for 40 hr and then monitored during gliding on glass coverslips via video microscopy. Images captured at one frame per second for 60 sec.
(MOV)

Video S2 DD-wild type parasites undergo normal helical gliding. DD-wild type parasites were treated with Shield-1 for 40 hr and then monitored during gliding on glass coverslips via video microscopy. Images captured at one frame per second for 60 sec.
(MOV)

Video S3 Parasites expressing mammalian-like TgACTI stall during circular gliding motility. DD-G200S parasites were treated with Shield-1 for 40 hr and then monitored during gliding on glass coverslips via video microscopy. Images captured at one frame per second for 60 sec.
(MOV)

Video S4 Parasites expressing mammalian-like TgACTI make incomplete circles during circular gliding motility. DD-G200S parasites were treated with Shield-1 for 40 hr and then monitored during gliding on glass coverslips via video microscopy. Images captured at one frame per second for 60 sec.
(MOV)

Video S5 Parasites expressing mammalian-like TgACTI move off track during circular gliding motility. DD-G200S parasites were treated with Shield-1 for 40 hr and then monitored during gliding on glass coverslips via video microscopy. Images captured at one frame per second for 60 sec.
(MOV)

Video S6 Parasites expressing mammalian-like TgACTI move more slowly and cover less distance during helical gliding motility. DD-G200S/K270M parasites were treated with Shield-1 for 40 hr and then monitored during gliding on glass coverslips via video microscopy. Images captured at one frame per second for 60 sec.
(MOV)
