# Supplementary figures and images for: Evolutionarily Divergent, Unstable Filamentous Actin Is Essential for Gliding Motility in Apicomplexan Parasites
Source: PLoS Pathog. 2011 Oct 6;7(10):e1002280. doi: 10.1371/journal.ppat.1002280 (PMC3188518; doi:10.1371/journal.ppat.1002280)

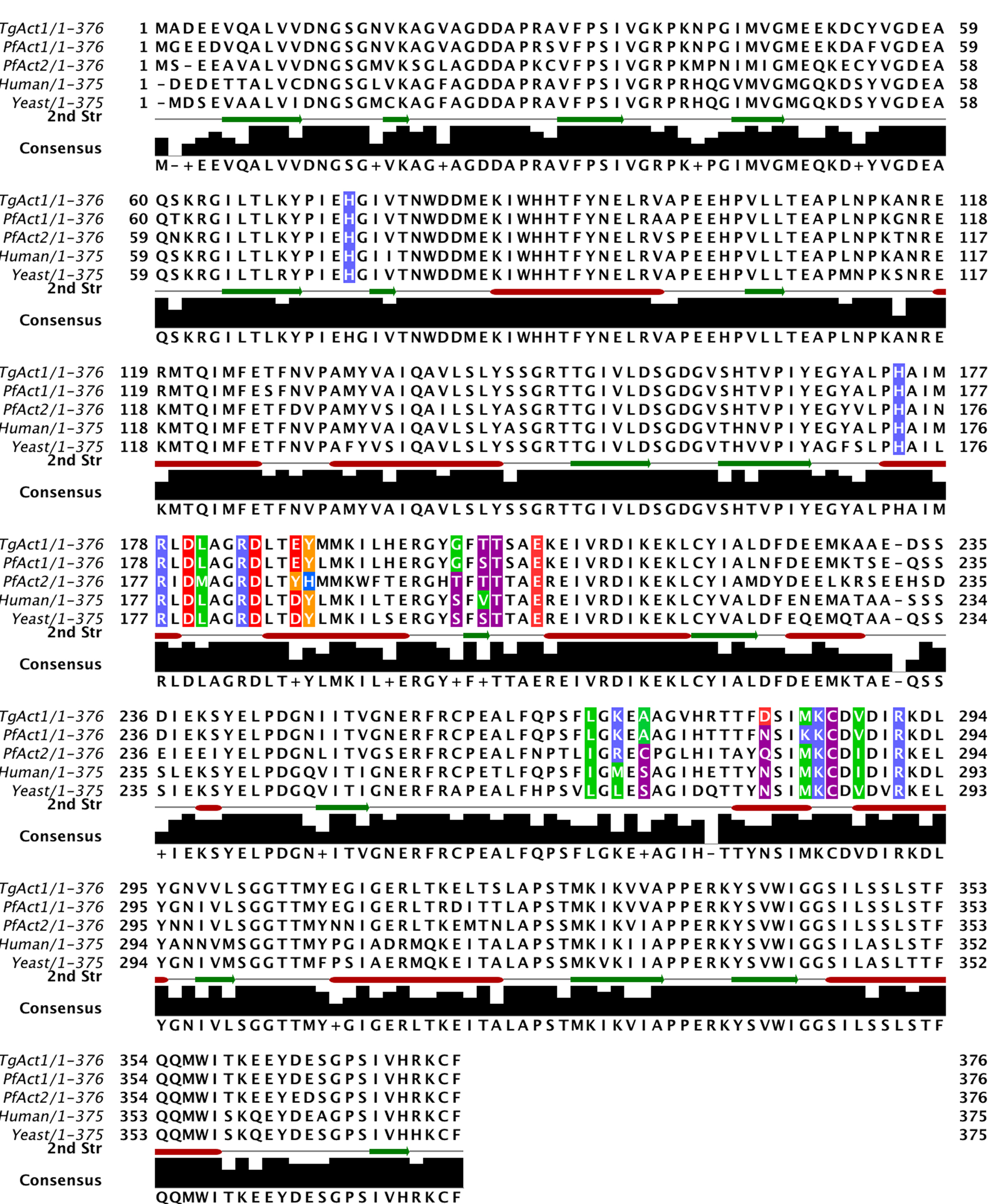

Supplement: Figure S1 — Sequence alignment for comparison of actins from Homo sapiens (muscle) (Human), Saccharomyces cerevisiae (Yeast), Toxoplasma gondii (TgACTI), Plasmodium falciparum (PfACTI or PfACTII). Residues that were mapped to within 4 Å of the phalloidin-binding site in muscle actin are highlighted. Color code: Blue - positive charged residues (including His), Red - negative charged, Green – hydrophobic, Purple - polar residues, Orange - aromatic. (TIF) [file ppat.1002280.s001.tif]

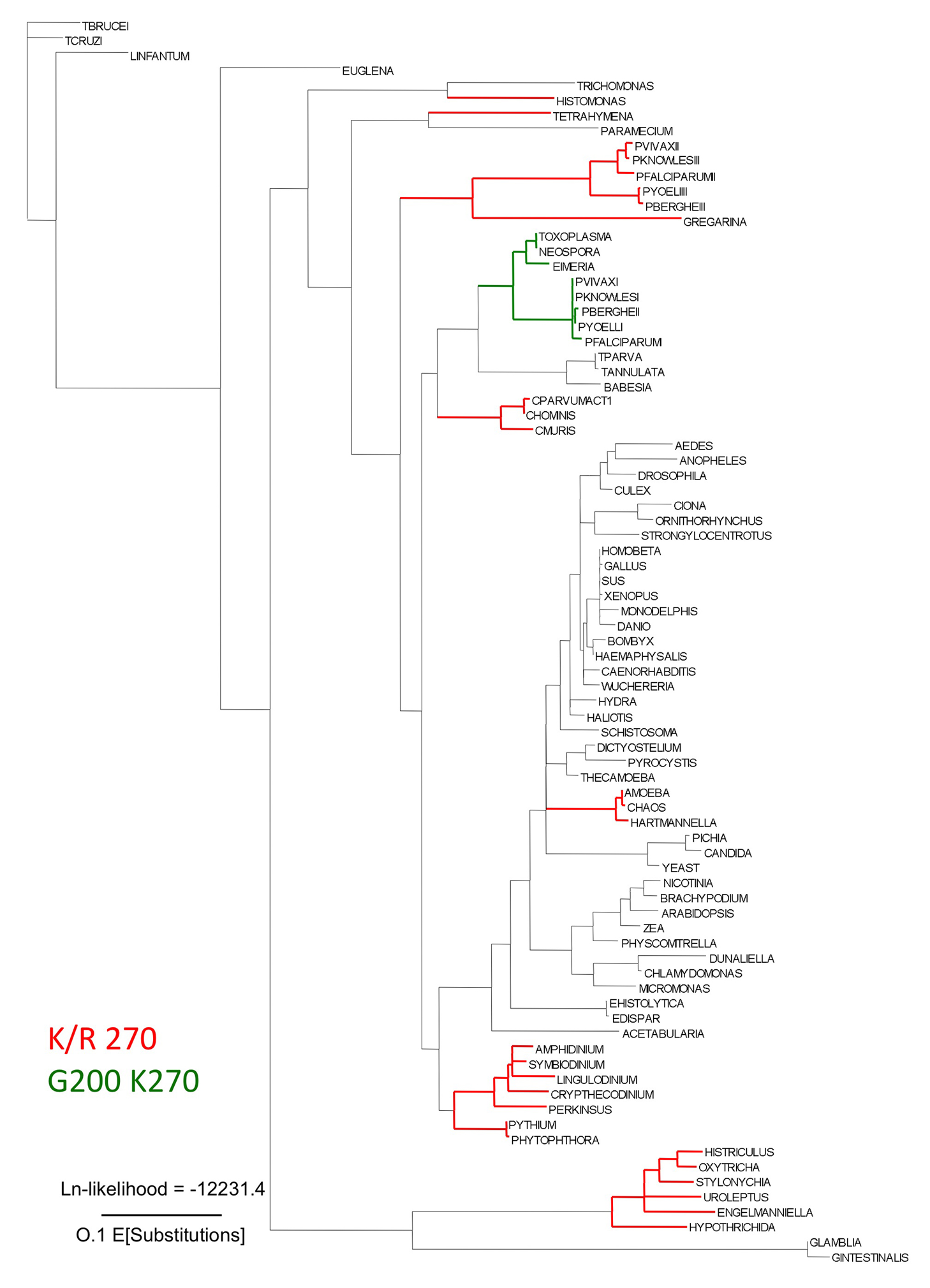

Supplement: Figure S2 — Maximum likelihood tree of diverse actins from a range of protists, fungi, plants and animals. The nexus output from the alignment file was the alignment file was imported into HyPhy [69] and used to generate a maximum likelihood tree under the HKY85 model with 100 bootstrap replicates. Red lines indicate taxa with K or R residues at 270, while green lines indicate taxa with G200 and K/R 270 (numbering based on T. gondii). See supplemental materials (Figure S5) for the alignment. (TIF) [file ppat.1002280.s002.tif]

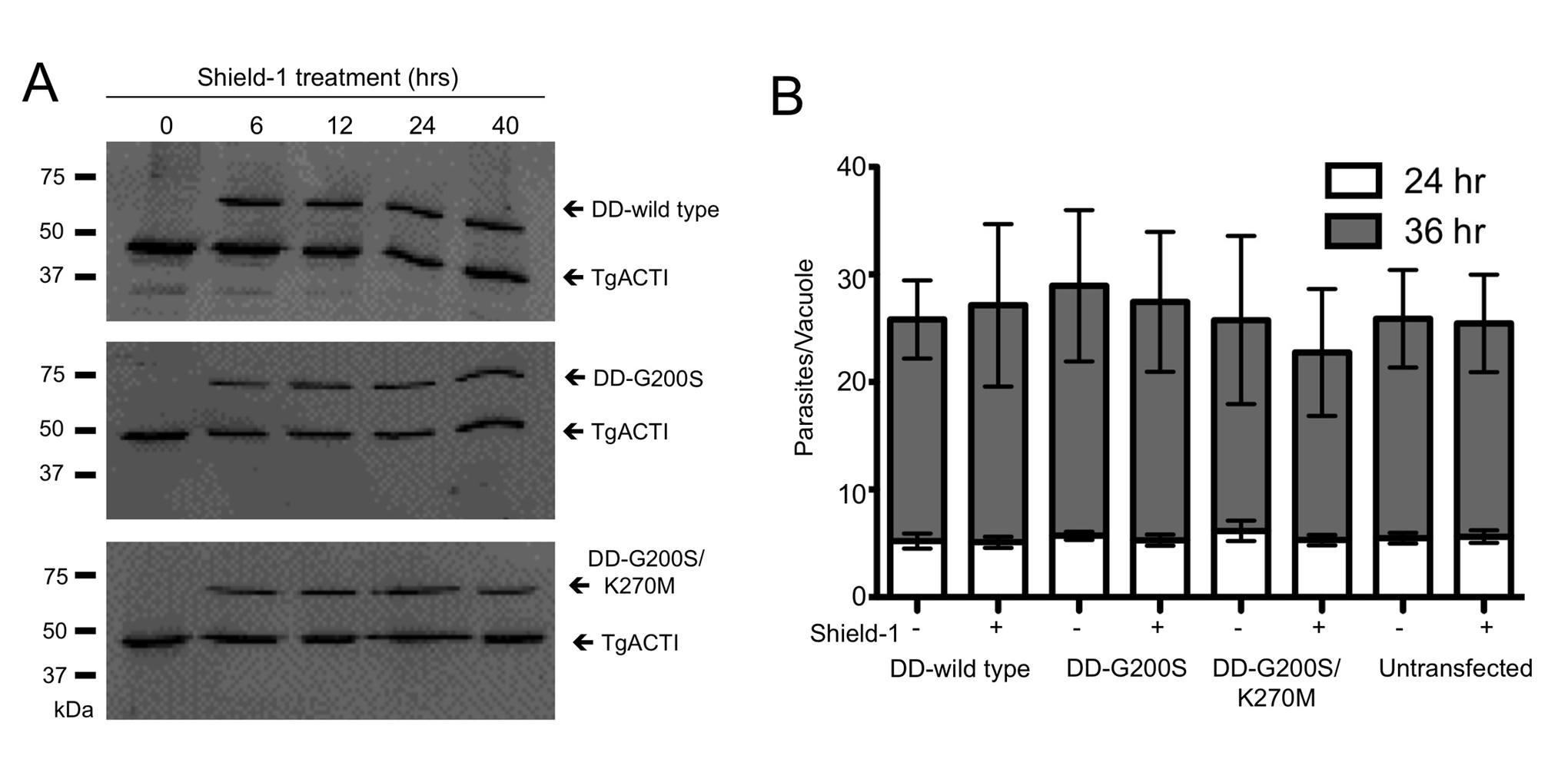

Supplement: Figure S3 — Time course of Shield treatment. A) Expression of DD-TgACTI fusion proteins following treatment ± Shield1 incubation for variable amounts of time (0, 6, 12, 24, 40 hr). All strains express the endogenous TgACTI while the fusion proteins (DD-wild type, DD-G200S or DD-G200S/K270M) were only expressed by the transfected strains in the presence of Shield-1. B) Replication of parasite strains expressing DD-TgACTI fusion proteins ± Shield1 at 24 and 36 hr. The numbers of parasites per vacuole were counted at the two time points. Mean ± S.D., n = 3 experiments. (TIF) [file ppat.1002280.s003.tif]

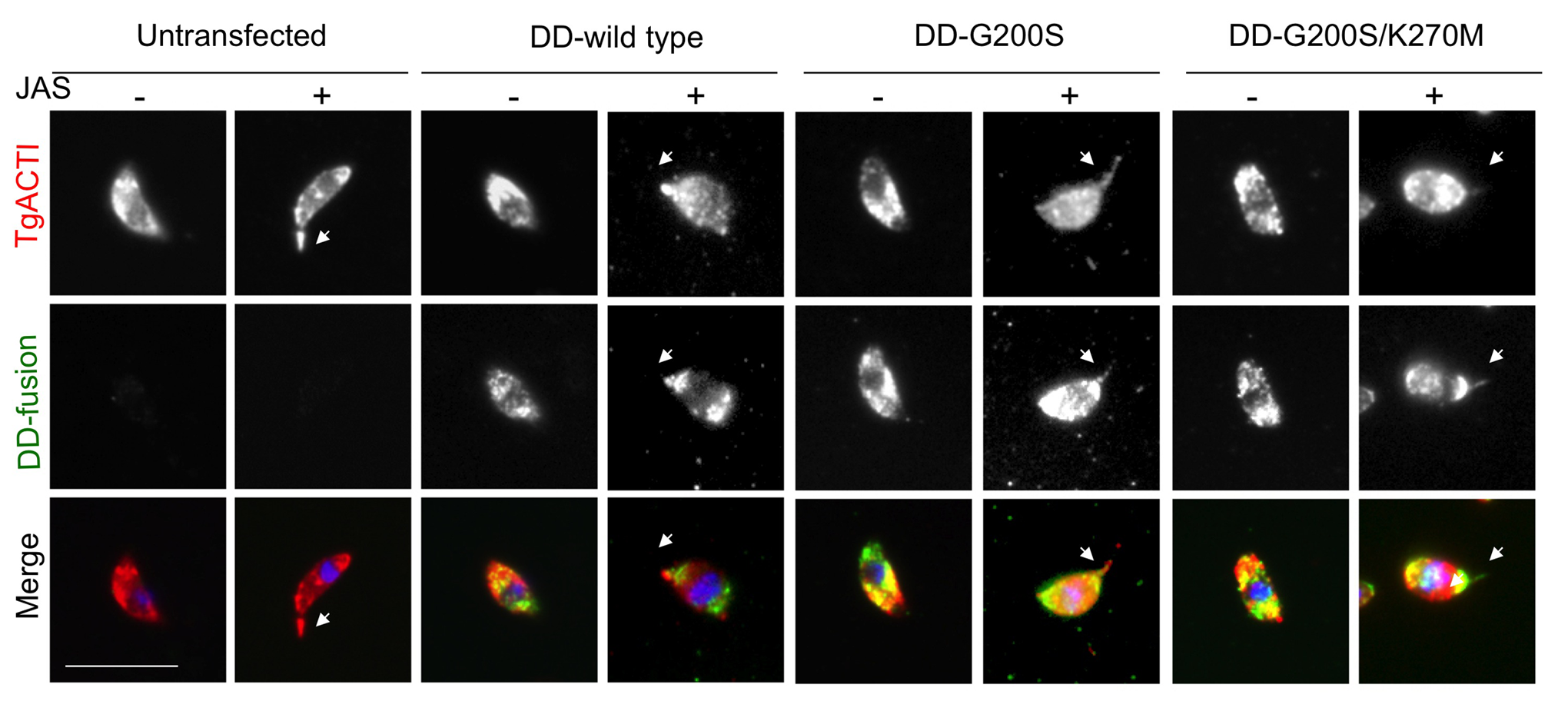

Supplement: Figure S4 — DD-TgACTI localization in JAS-induced actin protrusions. Expression of DD-tagged TgACTI alleles following treatment ± Shield-1 for 40 hr and JAS treatment (1 µM) for 15 min. Parasites were stained for immunofluorescence with anti-TgACTI (red) and anti-c-myc (green) to detect the DD-TgACTI fusion protein and compare localization in the JAS-induced actin protrusions. Scale bar, 5 µm. Arrowheads depict the JAS-induced actin protrusions. (TIF) [file ppat.1002280.s004.tif]

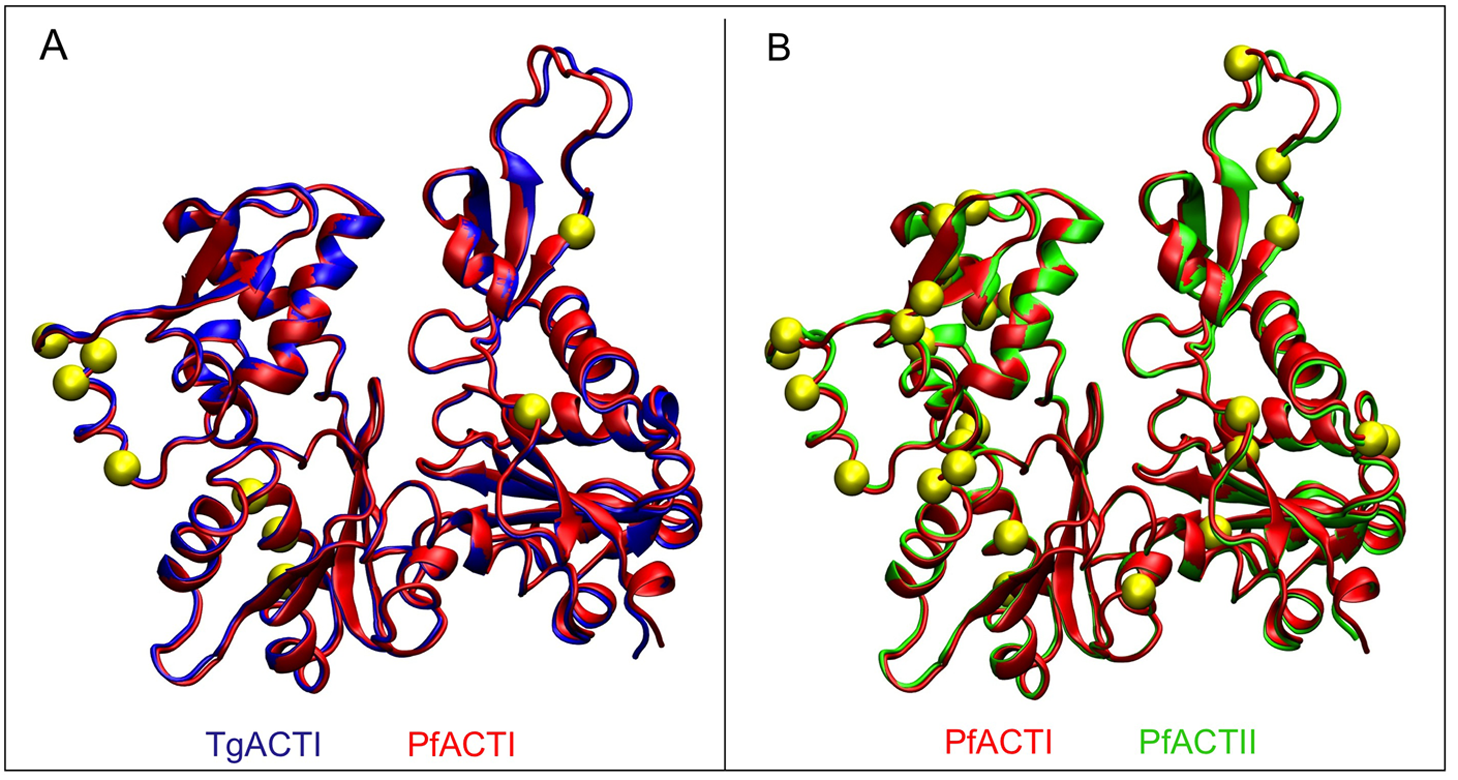

Supplement: Figure S6 — Enlarged parasite actin homology models. (A) Model of TgACTI (blue) mapped onto PfACTI (red) highlighting amino acid differences (yellow). (B) Model of PfACTI (red) mapped onto PfACTII (green) highlighting amino acid differences (yellow). (TIF) [file ppat.1002280.s006.tif]
