# Supplementary material for: Evolutionarily Divergent, Unstable Filamentous Actin Is Essential for Gliding Motility in Apicomplexan Parasites
Source: PLoS Pathog. 2011 Oct 6;7(10):e1002280. doi: 10.1371/journal.ppat.1002280 (PMC3188518; doi:10.1371/journal.ppat.1002280)

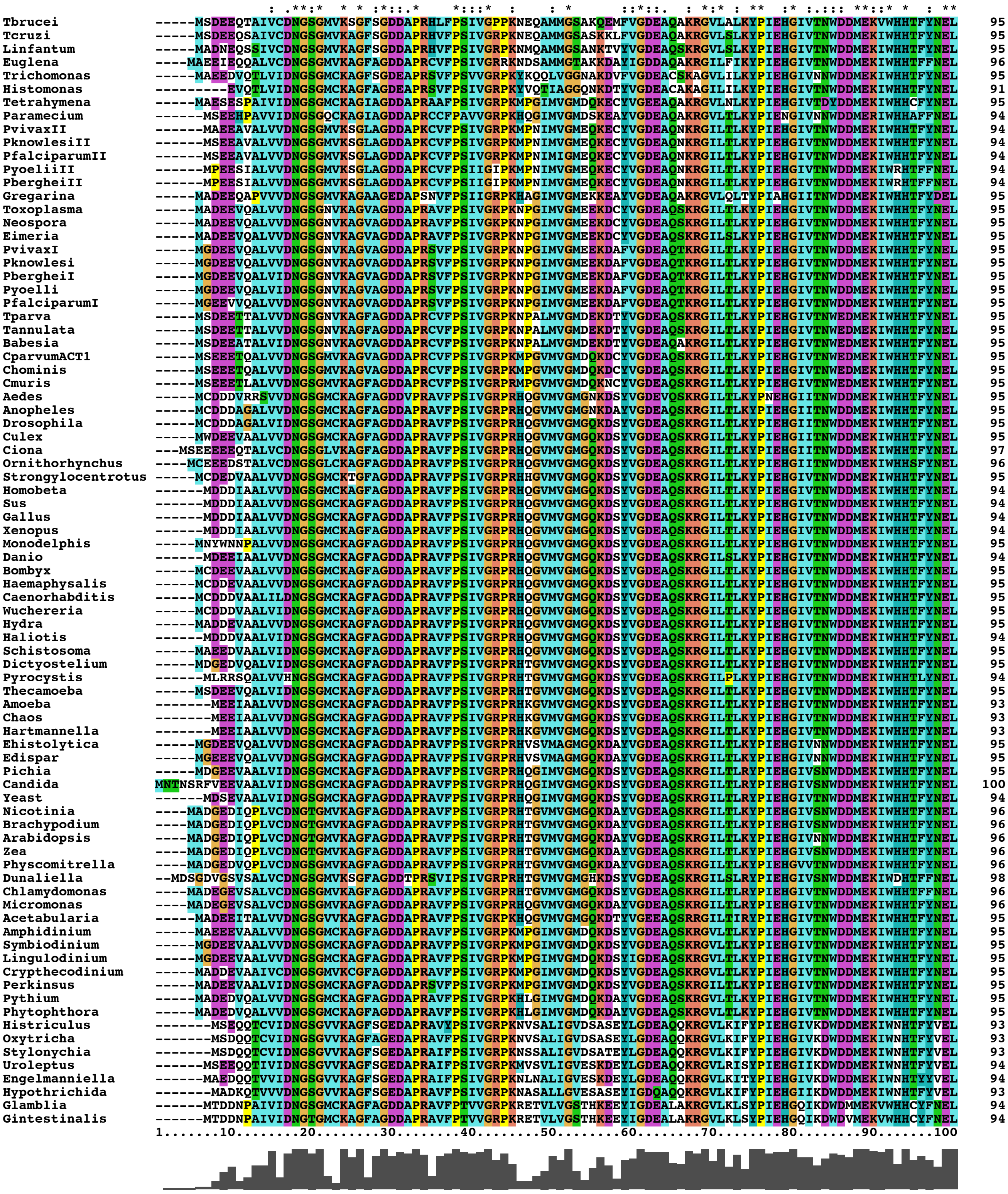
Tbrucei
Tcruzi
Linfantum
Euglena
Trichomonas
Histomonas
Tetrahymena
Paramecium
PvivaxII
PknowlesiII
PfalciparumII
PyoeliiII
PbergheiII
Gregarina
Toxoplasma
Neospora
Eimeria
PvivaxI
Pknowlesi
PbergheiI
Pyoelli
PfalciparumI
Tparva
Tannulata
Babesia
CparvumACT1
Chominis
Cmuris
Aedes
Anopheles
Drosophila
Culex
Ciona
Ornithorhynchus
Strongylocentrotus
Homobeta
Sus
Gallus
Xenopus
Monodelphis
Danio
Bombyx
Haemaphysalis
Caenorhabditis
Wuchereria
Hydra
Haliotis
Schistosoma
Dictyostelium
Pyrocystis
Thecamoeba
Amoeba
Chaos
Hartmannella
Ehistolytica
Edispar
Pichia
Candida
Yeast
Nicotinia
Brachypodium
Arabidopsis
Zea
Physcomitrella
Dunaliella
Chlamydomonas
Micromonas
Acetabularia
Amphidinium
Symbiodinium
Lingulodinium
Crypthecodinium
Perkinsus
Pythium
Phytophthora
Histriculus
Oxytricha
Stylonychia
Uroleptus
Engelmanniella
Hypothrichida
Glamblia
Gintestinalis
1.......10........20........30........40........50........60........70........80........90.......100

```
                    *  *  *  ***:.* *** ***::    *:..  *: *: ::*:*:**::***** .   **** * **:* *: :* .: * ::**:  *      :
Tbrucei             RVNPESHNVLLTEAPMNPKQNREKMTQIMFETFGVPAMYVGIQAVLSLYSSGRTTGIVLDAGDGVTHTVPIYEGYSLPHAIRRVDMAGRDLTEYLMKILM   195
Tcruzi              RVNPESHSVLLTEAPMNPKQNREKMTQIMFETFGVPAMYVGIQAVLSLYSSGRTTGIVLDAGDGVTHTVPIYEGYSLPHAIRRMDMAGRDLTEYLMKLLM   195
Linfantum           RVNPEQHNVLLTEAPMNPKQNREKMTQIMFETFNVPSLYIGIQAVLSLYSSGRTTGIVLDAGDGVTHTVPIYEGYSLPHAVRRVDMAGRDLTEYLMKIMM   195
Euglena             RVAPEDHPVLLTEAPMNPKSNREKMTQIMFETFNVPALYVSIQAVLSLYSSGRTTGIVLDCGDGVSHTVPIYEGYSLPHAVLRIDMAGRDLTDYMMKLLT   196
Trichomonas         RVDPTEHPVLLTEAPLNPKANREKMISLMFDTFNVPSFYVGIQAVLSLYSSGRTTGIVFDAGDGVSHTVPIYEGYSLPHAIMRLNLAGRDLTAWMVKLLT   195
Histomonas          RVDPAEHPVLLTEAPMNPKANREKMIQLQFETFNVPAFYVGIQAVLSLYSSGRTTGIVFDAGDGVSHTVPIYEGYSLPHAIMRINLAGRDLTAYLQKILN   191
Tetrahymena         RVTPEEHPCLLTEAPQNPKLNREKMTKTMFETFNVPSFYVAIQAVLSLYASGRTTGIVVDSGDGVTHTVPIYEGYALPHAILRIDLAGRELTEYCMKLLY   195
Paramecium          RVTPEDHPALLTEAPMNPKANREKMTQILFETFNVPSFYVAIQAVLSLYASGRTTGIVVDSGDGVSHTVPIYEGYALPHAVLRIDLAGRACTQYLVNILN   194
PvivaxII            RVSPEEHPVLLTEAPLNPKTNRERMTQIMFESFDVPAMYVSIQAILSLYASGRTTGIVLDSGDGVSHTVPIYEGYVLPHAINRIDMAGRDLTYHMMKLFT   194
PknowlesiII         RVSPEEHPVLLTEAPLNPKTNRERMTQIMFESFDVPAMYVSIQAILSLYASGRTTGIVLDSGDGVSHTVPIYEGYVLPHAINRIDMAGRDLTYHMMKLFT   194
PfalciparumII       RVSPEEHPVLLTEAPLNPKTNREKMTQIMFETFDVPAMYVSIQAILSLYASGRTTGIVLDSGDGVSHTVPIYEGYVLPHAINRIDMAGRDLTYHMMKWFT   194
PyoeliiII           RVSPEEHPVLLTEAPLNPKTNREKMTQIMFESFDVPAMYVSIQAILSLYASGRTTGIVLDSGDGVTHTVPIYEGYVLPHAINRTDMAGRDLTYYMMKLFT   194
PbergheiII          RVSPEEHPVLLTEAPLNPKTNREKMTQIMFESFDVPAMYVSIQAILSLYASGRTTGIVLDSGDGVTHTVPIYEGYVLPHAINRTDMAGRDLTYYMMKLFT   194
Gregarina           RISPEEHPVLLTEAPLNPKANREKMVQMMFENFSCPATYVSIQAVLSLYASGRTTGIVMDSGDGVSHTVPIYDGFALPHAVMRLDLAGRDLTEYMMTLLH   195
Toxoplasma          RVAPEEHPVLLTEAPLNPKANRERMTQIMFETFNVPAMYVAIQAVLSLYSSGRTTGIVLDSGDGVSHTVPIYEGYALPHAIMRLDLAGRDLTEYMMKILH   195
Neospora            RVAPEEHPVLLTEAPLNPKANRERMTQIMFETFNVPAMYVAIQAVLSLYSSGRTTGIVLDSGDGVSHTVPIYEGYALPHAIMRLDLAGRDLTEYMMKILH   195
Eimeria             RVAPEEHPVLLTEAPLNPKANRERMTQIMFETFNVPAMYVAIQAVLSLYSSGRTTGIVLDSGDGVSHTVPIYEGYALPHAMMRLDLAGRDLTEYMMTILH   195
PvivaxI             RAAPEEHPVLLTEAPLNPKGNRERMTQIMFESFNVPAMYVAIQAVLSLYSSGRTTGIVLDSGDGVSHTVPIYEGYALPHAIMRLDLAGRDLTEYLMKILH   195
Pknowlesi           RAAPEEHPVLLTEAPLNPKGNRERMTQIMFESFNVPAMYVAIQAVLSLYSSGRTTGIVLDSGDGVSHTVPIYEGYALPHAIMRLDLAGRDLTEYLMKILH   195
PbergheiI           RAAP-EHPVLLTEAPLNPKGNRERMTQIMFESFNVPAMYVAIQAVLSLYSSGRTTGIVLDSGDGVSHTVPIYEGYALPHAIMRLDLAGRDLTEYLMKILH   194
Pyoelli             RAAPEEHPVLLTEAPLNPKGNRERMTQIMFESFNVPAMYVAIQAVLSLYSSGRTTGIVLDSGDGVSHTVPIYEGYALPHAIMRLDLAGRDLTEYLMKILH   195
PfalciparumI        RAAPEEHPVLLTEAPLNPKGNRERMTQIMFESFNVPAMYVAIQAVLSLYSSGRTTGIVLDSGDGVSHTVPIYEGYALPHAIMRLDLAGRDLTEYLMKILH   195
Tparva              RIAPEEHPVLLTEAPMNPKANREKMTTIMFETHNVPAMYVAIQAVLSLYSSGRTTGIVLDSGDGVTHTVPIYEGYALPHAIMRLDLAGRDLTEFMQKILV   195
Tannulata           RIAPEEHPVLLTEAPMNPKANREKMTTIMFETHNVPAMYVAIQAVLSLYSSGRTTGIVLDSGDGVTHTVPIYEGYALPHAIMRLDLAGRDLTEFMQKILV   195
Babesia             RIAPEEHPVLLTEAPMNPKANREKMTTIMFETHNVPAMYVAIQAVLSLYSSGRTTGIVLDSGDGVTHTVPIYEGYALPHAIMRIDLAGRDLTEFMQKILA   195
CparvumACT1         RVAPEEHPVLLTEAPMNPKVNRERMTQIMFETFNVPAMYVNIQAVLSLYASGRTTGIVLDSGDGVSHTVPIYEGYAIPHAIMRLDLAGRDLTDFLMKILH   195
Chominis            RVAPEEHPVLLTEAPMNPKVNRERMTQIMFETFNVPAMYVNIQAVLSLYASGRTTGIVLDSGDGVSHTVPIYEGYALPHAIMRLDLAGRDLTDFLMKILH   195
Cmuris              RVAPEEHPVLLTEAPMNPKANRERMTQIMFETFNVPAMYVNIQAVLSLYASGRTTGIVLDSGDGVSHTVPIYEGYALPHAIMRLDLAGRDLTDYLMKILH   195
Aedes               RVAPEEHPVLLTEAPLNPKANREKMTQIMFETFNSPAMYVAIQAVLSLYASGRTTGIVLDSGDGVSHTVPIYEGYALPHAILRLDLAGRDLTDYLMKILT   195
Anopheles           RVAPEEHPVLLTEAPLNPKSNREKMTQIMFETFAAPAVYVAIQAVLSLYASGRTTGVVLDSGDGVSHTVPIYEGYALPHAILRMDLAGRDLTDYLMKILT   195
Drosophila          RVAPEEHPVLLTEAPLNPKANREKMTQIMFETFNSPAMYVAIQAVLSLYASGRTTGIVLDSGDGVSHTVPIYEGFALPHAILRLDLAGRDLTDYLMKILT   195
Culex               RVAPEEHPVLLTEAPLNPKANREKMTQIMFETFNSPAMYVAIQAVLSLYASGRTTGIVLDSGDGVSHTVPIYEGYALPHAILRLDLAGRDLTDYLMKILT   195
Ciona               RVAPEEHPVLLTEAPLNPKANREKMTQIMFETFNVPAMYVAIQAVLSLYASGRTTGIVLDSGDGVSHNVPIYEGYALPHAIMRLDLAGRDLTDYLMKILT   197
Ornithorhynchus     RVAPEEHPTLLTEAPLNPKANREKMTQIMFETFNVPAMYVAIQAVLSLYASGRTTGIVLDSGDGVTHNVPIYEGYALPHAIMRLDLAGRDLTDYLMKILT   196
Strongylocentrotus  RVAPEEHPVLLTEAPLNPKANREKMTQIMFETFNAPAMYVAIKAVLSLYASGRTTGIAXXXGDGVTHTVPIYEGYALPHAILRLDLAGRDLTDYLMKILT   195
Homobeta            RVAPEEHPVLLTEAPLNPKANREKMTQIMFETFNTPAMYVAIQAVLSLYASGRTTGIVMDSGDGVTHTVPIYEGYALPHAILRLDLAGRDLTDYLMKILT   194
Sus                 RVAPEEHPVLLTEAPLNPKANREKMTQIMFETFNTPAMYVAIQAVLSLYASGRTTGIVMDSGDGVTHTVPIYEGYALPHAILRLDLAGRDLTDYLMKILT   194
Gallus              RVAPEEHPVLLTEAPLNPKANREKMTQIMFETFNTPAMYVAIQAVLSLYASGRTTGIVMDSGDGVTHTVPIYEGYALPHAILRLDLAGRDLTDYLMKILT   194
Xenopus             RVAPEEHPVLLTEAPLNPKANREKMTQIMFETFNTPAMYVAIQAVLSLYASGRTTGIVMDSGDGVTHTVPIYEGYALPHAILRLDLAGRDLTDYLMKILT   194
Monodelphis         RVAPEEHPVLLTEAPLNPKANREKMTQIMFETFNTPAMYVAIQAVLSLYASGRTTGIVMDSGDGVTHTVPIYEGYALPHAILRLDLAGRDLTDYLMKILT   195
Danio               RVAPEEHPVLLTEAPLNPKANREKMTQIMFETFNTPAMYVAIQAVLSLYASGRTTGIVMDSGDGVTHTVPIYEGYALPHAILRLDLAGRDLTDYLMKILT   194
Bombyx              RVAPEEHPVLLTEAPLNPKANREKMTQIMFETFNTPAMYVAIQAVLSLYASGRTTGIVLDSGDGVSHTVPIYEGYALPHAILRLDLAGRDLTDYLMKILT   195
Haemaphysalis       RVAPEEHPVLLTEAPLNPKANREKMTQIMFETFNTPAMYVAIQAVLSLYASGRTTGIVLDSGDGVSHTVPIYEGYALPHAILRLDLAGRDLTDYLMKILT   195
Caenorhabditis      RVAPEEHPVLLTEAPLNPKANREKMTQIMFETFNTPAMYVAIQAVLSLYASGRTTGIVLDSGDGVTHTVPIYEGYALPHAILRLDLAGRDLTDYLMKILT   195
Wuchereria          RVAPEEHPVLLTEAPLNPKANREKMTQIMFETFNTPAMYVAIQAVLSLYASGRTTGIVLDTGDGVTHTVPIYEGYALPHAILRLDLAGRDLTDYLMKILT   195
Hydra               RVAPEEHPVLLTEAPLNPKANREKMTQIMFETFNSPAMYVAIQAVLSLYASGRTTGIVLDSGDGVSHTVPIYEGYALPHAIIRLDLAGRDLTDYLMKILT   195
Haliotis            RVAPEEHPVLLTEAPLNPKANREKMTQIMFETFNSPAMYVAIQAVLSLYASGRTTGIVLDSGDGVTHTVPIYEGYALPHAILRLDLAGRDLTDYMMKILT   194
Schistosoma         RVAPEEHPVLLTEAPLNPKANREKMTQIMFETFNVPAMYVAIQAVLSLYASGRTTGIVLDSGDGVTHTVPIYEGYALPHAILRLDLAGRDLTDYMMKILT   195
Dictyostelium       RVAPEEHPVLLTEAPLNPKANREKMTQIMFETFNTPAMYVAIQAVLSLYASGRTTGIVMDSGDGVSHTVPIYEGYALPHAILRLDLAGRDLTDYMMKILT   195
Pyrocystis          RVAPEEHPVLLTEAPLNPKANREKMTQIMFETFNTPAMYVAIQAVLSLYASGRTTGIVLDSGDGVSHTVPIYEGYALPHAILRLDLAGRDLTDYMMKILT   194
Thecamoeba          RVAPEEHPVLLTEAPLNPKANREKMTQIMFETFNTPAMYVAIQAVLSLYASGRTTGIVLDSGDGVTHTVPIYEGYALPHAILRLDLAGRDLTDYLMKILT   195
Amoeba              RVAPEEHPVLLTEAPLNPKANREKMTQIMFETFNVPAMYVAIQAVLSLYASGRTTGIVCDIGDGVCHTVPIYEGYALPHAIARLDLAGRDLTDYCMKILT   193
Chaos               RVAPEEHPVLLTEAPLNPKANREKMTQIMFETFNVPAMYVAIQAVLSLYASGRTTGIVCDIGDGVCHTVPIYEGYALPHAIARLDLAGRDLTDYCMKILT   193
Hartmannella        RVAPEEHPVLLTEAPLNPKANREKMTQIMFETFNVPAMYVAIQAVLSLYASGRTTGIVLDIGDGVCHTVPIYEGYALPHAIARLDLAGRDLTDYCMKILT   193
Ehistolytica        RVAPEEHPVLLTEAPMNPKANREKMTQIMFETFNTPAMYVGIQAVLSLYASGRTTGIVMDSGDGVSHTVPIYEGFSLPHAILRLDLAGRDLTDYLMKILT   195
Edispar             RVAPEEHPVLLTEAPMNPKANREKMTQIMFETFNTPAMYVGIQAVLSLYASGRTTGIVMDSGDGVSHTVPIYEGFSLPHAILRLDLAGRDLTDYLMKILT   195
Pichia              RVAPEEHPVLLTEAPMNPKSNREKMTQIMFETFNVPAFYVSIQAVLSLYSSGRTTGIVLDSGDGVTHVVPIYAGFSLPHGILRIDLAGRDLTDYLMKILS   195
Candida             RVAPEEHPVLLTEAPMNPKSNREKMTQIMFETFNVPAFYVSIQAVLSLYSSGRTTGIVLDSGDGVTHVVPIYAGFSLPHGILRIDLAGRDLTDYLMKILS   200
Yeast               RVAPEEHPVLLTEAPMNPKSNREKMTQIMFETFNVPAFYVSIQAVLSLYSSGRTTGIVLDSGDGVTHVVPIYAGFSLPHAILRIDLAGRDLTDYLMKILS   194
Nicotinia           RVAPEEHPVLLTEAPLNPKANREKMTQIMFETFNTPAMYVAIQAVLSLYASGRTTGIVLDSGDGVSHTVPIYEGYALPHAILRLDLAGRDLTDHLMKILT   196
Brachypodium        RVAPEEHPVLLTEAPLNPKANREKMTQIMFETFNTPAMYVAIQAVLSLYASGRTTGIVLDSGDGVSHTVPIYEGYALPHAILRLDLAGRDLTDYMMKILT   196
Arabidopsis         RVAPEEHPILLTEAPLNPKANREKMTQIMFETFNAPAMYVAIQAVLSLYASGRTTGIVLDSGDGVSHTVPIYEGYALPHAILRLDLAGRDLTDALMKILT   196
Zea                 RVAPEEHPVLLTEAPLNPKANREKMTQIMFETFNVPAMYVAIQAVLSLYASGRTTGIVLDSGDGVSHTVPIYEGYALPHAILRLDLAGRDLTDSLMKILT   196
Physcomitrella      RVAPEEHPVLLTEAPLNPKANREKMTQIMFETFNVPAMYVAIQAVLSLYASGRTTGIVLDSGDGVTHTVPIYEGYALPHAILRLDLAGRDLTDALMKILT   196
Dunaliella          RVAPEEHPVLLTEAPLNPKGNREKMTQIMFETFNVPAMYVAIQAVLSLYASGRTTGIVLDSGDGVTHTVPIYEGYALPHAILRLDLAGRDLTDYLMKILM   198
Chlamydomonas       RVAPEEHPVLLTEAPLNPKANREKMTQIMFETFNVPAMYVAIQAVLSLYASGRTTGIVLDSGDGVTHTVPIYEGYALPHAILRLDLAGRDLTDYLMKILM   196
Micromonas          RVAPEEHPVLLTEAPLNPKANREKMCQIMFETFNVPAMYVAIQAVLSLYASGRTTGIVLDCGDGVSHTVPIYEGYALPHAILRLDLAGRDLTDYLMKILT   196
Acetabularia        RVAPEEHPVLLTEAPMNPKSNREKLTQIMFETFNVPAMYVSIQAVLSLYASGRTTGIVLDSGDGVSHTVPIYEGYALPHAILRLDLAGRDLTDYLTKLLM   195
Amphidinium         RVAPEEHPVLLTEAPLNPKANRERMTQIMFETFNVPAMYVAIQAVLSLYASGRTTGIVMDSGDGVSHTVPIYEGYALPHAILRLDLAGRDLTEYLMKILT   195
Symbiodinium        RXAPEEHPVLLTEAPLNPKANRERMTQIMFETFNVPAMYVAIQAVLSLYASGRTTGIVMDSGDGVSHTVPIYEGYALPHAILRLDLAGRDLTEYMMKILT   195
Lingulodinium       RVAPEEHPVLLTEAPLNPKANRERMTQIMFETFNVPAMYVAIQAVLSLYASGRTTGIVMDSGDGVSHTVPIYEGYALPHAILRLDLAGRDLTDYLMKILT   195
Crypthecodinium     RVAPEEHPVLLTEAPLNPKANRERMTQIMFETFNVPAMYVAIQAVLSLYASGRTTGIVMDSGDGVSHTVPIYEGYALPHAILRLDLAGRDLTEYMMKILT   195
Perkinsus           RVSPEGHPVLLTKAPLNPKANRERMTQLMFETFNVPAMYVQIQAVLSLYASGRTTGIVMDSGDGVSHTVPIYEGYALPHAIQRLDLAGRDLTEFMMKILT   195
Pythium             RVAPEEHPVLLTEAPLNPKANRERMTQIMFETFNVPAMYVNIQAVLSLYASGRTTGCVLDSGDGVSHTVPIYEGYALPHAIVRLDLAGRDLTDYMMKILT   195
Phytophthora        RVAPEEHPVLLTEAPLNPKANRERMTQIMFETFNVPAMYVNIQAVLSLYASGRTTGCVLDSGDGVSHTVPIYEGYALPHAIVRLDLAGRDLTDYMMKILT   195
Histriculus         RVSPDEHPVLLTEAPLNPKVNREKMTQIMFETFNVPALYVAIQAVLSLYSAGRTTGIVCDAGDGVTHTVPIYEGFSIPHAVSRIQLAGRDLTTFMAKLLT   193
Oxytricha           RVQPDEHPILLTEAPLNPKTNREKMTQIMFETFNVPALYVAIQAVLSLYSAGRTTGIVCDAGDGVTHTVPIYEGFSIPHAVSRIQLAGRDLTTFMAKLLT   193
Stylonychia         RVSPDEHPVLLTEAPLNPKINREKMTQIMFETFNVPALYVAIQAVLSLYSAGRTTGIVCDAGDGVTHTVPIYEGFSIPHAVQRIQLAGRDLTMFMAKLLT   193
Uroleptus           RVSPDEHPVLLTEAPLNPKVNREKMTQIMFETFNVPALYVAIQAVLSLYSAGRTTGIVCDAGDGVTHTVPIYEGFSIPHAVSRIQLAGRDLTQFMAKLLT   194
Engelmanniella      RVSPDEHPVLLTEAPLNPKVNREKMTQIMFETFNVPSLYVAIQAVLSLYSAGRTTGIVCDSGDGVTHTVPIYEGFSIPXAISRIQLAGRDLTTFMAKLLT   194
Hypothrichida       RVQPDEHPILLTEAPLNPKVNREKMTQIMFETFNVPALYVAIQAVLSLYSAGRTTGIVCDAGDGVTHTVPIYEGFSIPHAVSRIQLAGRDLTTFMAKLLT   193
Glamblia            RAQPSDHAVLLTEAPKNPKANREKICQIMFETFAVPAFYVQVQAVLALYSSGRTTGIVIDTGDGVTHTVPVYEGYSLPHAVLRSEIAGKELTDFCQINLQ   194
Gintestinalis       RAQPSDHAVLLTEPPKNPKANREKLCQIMFETFAVPAFYVQVQAVLALYSSGRTTGIVIDTGDGVTHTVPVYEGYSLPHAVLRSEIAGKELTDFCQINLQ   194
                    .......110.......120.......130.......140.......150.......160.......170.......180.......190.......200
```

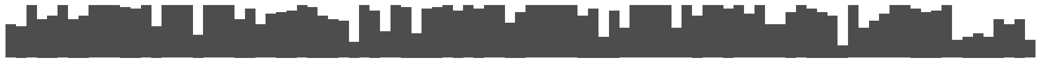

```
                      *  * ::*: **:*: **   : * : :                : ****  : :   *::  :        .      .    :.::  **:*:* :
Tbrucei             ETGMTFTTSAQKEIVRNIKEQLCYVALDFDEEMTNSAKS-VSEEP-FELPDGNVMQVGNQRFRCPQALFKPALIGLDEAPGFHEMTFQSINKCDIDVRRD   293
Tcruzi              ESGMTFTTSAEKEIVRNVKEQLCYVALDFDEEVTNSAKT-VNEEP-FELPDGTIMQVGNQRFRCPEALFKPMLIGLDEAPGFHEMTFQSINKCDIDVRRD   293
Linfantum           ETGMTFTTTAEKEIVRNVKEQLCYVALDFEEEMTNSAKS-ANEEA-FELPDGNVMMVGNQRFRCPEVLFKPSLIGLDEAPGFPEMVYQSINKCDIDVRRE   293
Euglena             ERGLSFTTSAEREIVRDVKEKLCYVALDFDEEMSLATSSSSVEKE-YELPDGNIIQVGSERFRCPEVLMKPSMIGLEACG-VHETTFNSINKCDIDVRKD   294
Trichomonas         ERGNAFNTTAEKEIVRDIKEKLCYVALDFDAEMEKAATDSSINVN-YTLPDGNVITIGNERFRCPEMLFKPYFDGM-EYDGIDKTLFDSIMKCDIDVRKD   293
Histomonas          ERGYTFTTSAEKEIVRDIKEKHAYVALDFDEEMNKAATTSECDIS-YTLPDGNVITIANERFRCPELLFKPHFNGF-EFEGIDQTLFNSIMACDIDVRKD   289
Tetrahymena         EIGLNFSSTAEREIIRDIKEKLCYVAIDYEAELKAYKESSQNDKS-YELPDGNTITVQDERFRCPELLFKPAFIGK-EFPGIHELTFNSIMKCDVDVRKD   293
Paramecium          ELGVSFTSSAEMEIVRDMKEKLCYVALDYEEELKKYKESAANNRP-YELPDGNVVVIQNQRFRCPELLFKPAFIGL-EVSGLHELTFKSIMKCDIDVRKD   292
PvivaxII            ERGHTFTTTAEREIVRDIKEKLCYIALDYDEELKKSEEHADQIEEIYELPDGNLITVGSERFRCPEALFNPTLIGR-ECPGLHITAYQSIMKCDIDIRKE   293
PknowlesiII         ERGHTFTTTAEREIVRDIKEKLCYIALDYDEELKKSEEHSDQIEEIYELPDGNLITVGSERFRCPEALFNPTLIGR-ECPGLHITAYQSIMKCDIDIRKE   293
PfalciparumII       ERGHTFTTTAEREIVRDIKEKLCYIAMDYDEELKRSEEHSDEIEEIYELPDGNLITVGSERFRCPEALFNPTLIGR-ECPGLHITAYQSIMKCDIDIRKE   293
PyoeliiII           ERGYTFTTTAEREIVRDIKEKLCYIALDYDEELKKSEERTEQVEEMYELPDGNLITVGSERFRCPEALFNPSLIGR-ECPGLHITAYQSIMKCDIDIRKE   293
PbergheiII          ERGYTFTTTAEREIVRDIKEKLCYIALDYDEELKKSEERTEEVEEMYELPDGNLITVGSERFRCPEALFNPSLIGR-ECPGLHITAYQSIMKCDIDIRKE   293
Gregarina           LRGHAFTTSAEKEIVRDIKEKLCYIALEFDEELKKAEE-SDEVEKTYELPDGNVISVGKERFQTPEALFQPSLIGR-EAQGVHHTTFQSVMKCDVDIRKD   293
Toxoplasma          ERGYGFTTSAEKEIVRDIKEKLCYIALDFDEEMKAAEDSSDIEKS-YELPDGNIITVGNERFRCPEALFQPSFLGK-EAAGVHRTTFDSIMKCDVDIRKD   293
Neospora            ERGYGFTTSAEKEIVRDIKEKLCYIALDFDEEMKAAEDSSDIEKS-YELPDGNIITVGNERFRCPEALFQPSFLGK-EAAGVHRTTFDSIMKCDVDIRKD   293
Eimeria             ERGYGFTTTAEKEIVRDIKEKLCYIALDFDEEMK--EDSSDIEKS-YELPDGNIITVGNGRLRCPEALFQPSFLGK-EASGIHRTTFDSIMKCDVDIRKD   291
PvivaxI             ERGYGFSTSAEKEIVRDIKEKLCYIALNFDEEMKTSEQSSDIEKS-YELPDGNIITVGNERFRCPEALFQPSFLGK-EAAGIHTTTFNSIKKCDVDIRKD   293
Pknowlesi           ERGYGFSTSAEKEIVRDIKEKLCYIALNFDEEMKTSEQSSDIEKS-YELPDGNIITVGNERFRCPEALFQPSFLGK-EAAGIHTTTFNSIKKCDVDIRKD   293
PbergheiI           ERGYGFSTSAEKEIVRDIKEKLCYIALNFDEEMKTSEQSSDIEKS-YELPDGNIITVGNERFRCPEALFQPSFLGK-EAAGIHTTTFNSIKKCDVDIRKD   292
Pyoelli             ERGYGFSTSAEKEIVRDIKEKLCYIALNFDEEMKTSEQSSDIEKS-YELPDGNIITVGNERFRCPEALFQPSFLGK-EAAGIHTTTFNSIKKCDVDIRKD   293
PfalciparumI        ERGYGFSTSAEKEIVRDIKEKLCYIALNFDEEMKTSEQSSDIEKS-YELPDGNIITVGNERFRCPEALFQPSFLGK-EAAGIHTTTFNSIKKCDVDIRKD   293
Tparva              ERGFSFTTTAEKEIVRDIKEKLCYIALDFDEEMTTSSSSSEVEKS-YELPDGNIITVGNERFRCPEVLFQPTFIGM-EAPGIHTTTYNSIVRCDVDIRKD   293
Tannulata           ERGFSFTTTAEKEIVRDIKEKLCYIALDFDDEMTTSSSSSEVEKS-YELPDGNIITVGNERFRCPEVLFQPTFIGM-EAPGIHTTTYNSIVRCDVDIRKD   293
Babesia             ERGFTFTTTAEKEIVRDIKEKLCYIALDFEEEMNSSASSSEIEKS-YELPDGNIITVGNERFRCPEVLFQPSFIGQ-ESAGIHTTTFNSIARCDVDIRKD   293
CparvumACT1         DRGYSFTTTAEREIVRDIKEKLCYIALDYEEEMKKSQESSELEKT-YELPDGHVITVGSERFRCPEALFQPGFLGK-EAVGIGETTFQSIMKCDLDIRKD   293
Chominis            DRGYSFTTTAEREIVRDIKEKLCYIALDYEEEMKKSQESSELEKT-YELPDGHVITVGSERFRCPEALFQPGLLGK-EAVGIGETTFQSIMKCDLDIRKD   293
Cmuris              DRGYSFTTTAEREIVRDIKEKLCYIALDYEDELKKAQESSEIEKT-YELPDGNVITVGSERFRCPEALFQPNLLGK-EASGVGETTFQSIMKCDLDIRKD   293
Aedes               ERGYSFTTTAEREIVRDIKEKLCYVALDFEQEMATAAASTSLEKS-YELPDGQVITIGNERFRAQKLSSSHPSLGM-ESCGIHETVYNSIMKCDVDIRKD   293
Anopheles           ERGYSFTTTAEREIVRDIKEKLCYVALDFEQEMQAAAASSSSEKS-YELPDGQVITIGNERFRAPEALFQPSFLGM-ESTGIHETVYNSIMRCDVDIRKD   293
Drosophila          ERGYTFTTTAEREIVRDTKEKLCYVALDFEDEMATAAASTSLEKS-YELPDGQVITIGNERFRCPEALFQPSFLGM-ESCGIHETVYNSIMKCDVDIRKD   293
Culex               ERGYSFTTTAEREIVRDIKEKLCYVALDFEQEMATAAASTSLEKS-YELPDGQVITIGNERFRCPEALFQPSFLGM-ESAGIHETVYNSIMKCDVDIRKD   293
Ciona               ERGYSFVTTAEREIVRDIKEKLCYVALDFEQEMATAASSSGLEKS-YELPDGQVITIGNERFRCPETMFQPSFIGM-ESSGVHETTYNSIMKCDIDIRKD   295
Ornithorhynchus     ERGYSFVTTAEREIVRDIKEKLCYVALDFENEMATAATSSSLEKS-YELPDGQVITIGNERFRCPETLFQPSFIGM-ESAGIHETTYNSIMKCDIDIRKD   294
Strongylocentrotus  ERGYSFTTTAEREIVRDIKEKLCYTALNFEREMATAAASSSLEKS-YELPDGQVITIGNERFRCPETLFQPAFIGM-ESAGIHETTYNSIMKCDIDIRKD   293
Homobeta            ERGYSFTTTAEREIVRDIKEKLCYVALDFEQEMATAASSSSLEKS-YELPDGQVITIGNERFRCPEALFQPSFLGM-ESCGIHETTFNSIMKCDVDIRKD   292
Sus                 ERGYSFTTTAEREIVRDIKEKLCYVALDFEQEMATAASSSSLEKS-YELPDGQVITIGNERFRCPEALFQPSFLGM-ESCGIHETTFNSIMKCDVDIRKD   292
Gallus              ERGYSFTTTAEREIVRDIKEKLCYVALDFEQEMATAASSSSLEKS-YELPDGQVITIGNERFRCPEALFQPSFLGM-ESCGIHETTFNSIMKCDVDIRKD   292
Xenopus             ERGYSFTTTAEREIVRDIKEKLCYVALDFEQEMATAASSSSLEKS-YELPDGQVITIGNERFRCPEALFQPSFLGM-ESCGIHETTYNSIMKCDVDIRKD   292
Monodelphis         ERGYSFTTTAEREIVRDIKEKLCYVALDFEQEMATAASSSSLEKS-YELPDGQVITIGNERFRCPEALFQPSFLGM-ESCGIHETTFNSIMKCDVDIRKD   293
Danio               ERGYSFTTTAEREIVRDIKEKLCYVALDFEQEMGTAASSSSLEKS-YELPDGQVITIGNERFRCPEALFQPSFLGM-ESCGIHETTFNSIMKCDVDIRKD   292
Bombyx              ERGYSFTTTAEREIVRDIKEKLCYVALDFEQEMATAASSSSLEKS-YELPDGQVITIGNERFRCPEALFQPSFLGM-EANGIHETTYNSIMKCDVDIRKD   293
Haemaphysalis       ERGYSFTTTAEREIVRDIKEKLCYVALDFEQEMATAASSSSLEKS-YELPDGQVITIGNERFRCPEALFQPSFLGM-ESCGIHETTYNSIMKCDVDIRKD   293
Caenorhabditis      ERGYSFTTTAEREIVRDIKEKLCYVALDFEQEMATAASSSSLEKS-YELPDGQVITVGNERFRCPEALFQPSFLGM-ESAGIHETSYNSIMKCDIDIRKD   293
Wuchereria          ERGYSFTTTAEREIVRDIKEKLCYVALDFEQEMATAASSSSLEKS-YELPDGQVITIGNERFRCPEALFQPSFLGM-ESTGIHETTYNSIMKCDVDIRKD   293
Hydra               ERGYSFTTTAEREIVRDIKEKLSYVALDFEQEMQTAASSSSLEKS-YELPDGQVITIGNERFRCPETLFQPSFIGM-ESAGIHETTYNSIMKCDVDIRKD   293
Haliotis            ERGYSFTTTAEREIVRDIKEKLAYVALDFEQEMQTAASSSSLEKS-YELPDGQVITIGNERFRCPEALFQPSFLGM-ESAGIHETTYNSIMKCDVDIRKD   292
Schistosoma         ERGYSFTTTAEREIVRDIKEKLCYVALDFEQEMGTAASSSALEKS-YELPDGQVITIGNERFRCPEALFQPSFLGM-EASGIHETTYNSIMKCDVDIRKD   293
Dictyostelium       ERGYSFTTTAEREIVRDIKEKLAYVALDFEQEMATAASSSALEKS-YELPDGQVITIGNERFRCPEALFQPSFLGM-ESAGIHETTYNSIMKCDVDIRKD   293
Pyrocystis          ERGYSFTTTAEREIVRDIKEKLAYVALDFEQEMQTAASSSALEKS-YELPDGQVITIGNERFRCPEALFQPSFLGM-ESAGVHETTYNSIMKCDVDIRKD   292
Thecamoeba          ERGYSFTTTAEREIVRDIKEKLAYVALDFEQEMQTAASSSSLEKS-YELPDGQVITIGNERFRCPEALFQPSFLGM-EAAGIHETTYNSIMKCDVDIRKD   293
Amoeba              ERGYAFTTTAEREIVRDIKEKLCYVALDFDAEMDTAATSSTIEKS-YELPDGQVITVGNERFRTPEALFQPSFLGR-ESAGIHEATYNSIMKCDIDIRKD   291
Chaos               ERGYAFTTTAEREIVRDIKEKLCYVALDFDAEMDTAATSSTIEKS-YELPDGQVITVGNERFHTPEALFQPSFLGR-ESAGIHEATYNSIMKCDIDIRKD   291
Hartmannella        ERGYAFTTTAEREIVRDIKEKLCYVALDFDAEMDTAASSSTIEKS-YELPDGQVITVGNERFRTPEALFQPSFLGR-ESAGIHEATYNSIMKCDIDIRKD   291
Ehistolytica        ERGYAFTTTAEREIVRDIKEKLCYVAEDFNEEMQKAASSSELEKS-YELPDGQVITVGNERFRCPEALFQPSFLGM-ECNGIHETTYNSIMKCDVDIRKD   293
Edispar             ERGYAFTTTAEREIVRDIKEKLCYVAEDFNEEMQKAASSSELEKS-YELPDGQVITVGNERFRCPEALFQPSFLGM-ECNGIHETTYNSIMKCDVDIRKD   293
Pichia              ERGYTFSTTAEREIVRDIKEKLCYVALDFEQEMQTSSQSSAIEKS-YELPDGQVITIGNERFRAAEALFRPADLGL-EAAGIDQTTFNSIMKCDVDVRKE   293
Candida             ERGYTFSTTAEREIVRDIKERLCYVALDFEQEMQTSSQSSAIEKS-YELPDGQVITIGNERFRAPEALFRPADLGL-EAAGIDQTTFNSIMKCDLDVRKE   298
Yeast               ERGYSFSTTAEREIVRDIKEKLCYVALDFEQEMQTAAQSSSIEKS-YELPDGQVITIGNERFRAPEALFHPSVLGL-ESAGIDQTTYNSIMKCDVDVRKE   292
Nicotinia           ERGYSFTTTAEREIVRDVKEKLSYIALDFEQEMETSKTSSSVEKS-YELPDGQVITIGAERFRCPEVLFQPSMIGM-EAAGIHETTYNSIMECDVDIRKD   294
Brachypodium        ERGYSFTTTAEREIVRDMKEKLSYIALDYDQEMETSKTSSSVEKS-YELPDGQVITIGAERFRCPEVLFQPSFIGM-EAAGIHETTYNSIMKCDVDIRKD   294
Arabidopsis         ERGYSFTTTAEREIVRDIKEKLCYIALDYEQELETAKTSSSVEKN-YELPDGQVITIGSERFRCPEVLYQPSMIGM-ENAGIHETTYNSIMKCDVDIRKD   294
Zea                 ERGYSFTTTAEREIVRDIKEKLAYVALDYDQELENAKSSSSVEKS-YELPDGQVITIGAERFRCPEVLFQPSFIGM-EAPGIHETTYNSIMKCDVDIRKD   294
Physcomitrella      ERGYSFTTTAEREIVRDMKEKLAYVAIDFEQELDTARSSSSLEKS-YELPDGQVITIGAERFRCPEVLFNPSLIGM-EAAGIHETTYNSIMKCDVDIRKD   294
Dunaliella          ERGYSFTTTAEREIVRDIKEKLAYVALDFEQEMATAQSSSALDKN-YELPDGQTITIGNERFRCPEVMFNPNTVGM-EAVGVHDTTFNSIMKCDVDIRKD   296
Chlamydomonas       ERGYSFTTTAEREIVRDIKEKLCYVALDFEQEMATALSSSALEKT-YELPDGQMITIGNERFRCPEVLFNPNMIGM-EAVGIHDTTFNSIMKCDVDIRKD   294
Micromonas          ERGYSFTTSAEREIVRDIKEKLAYVALDFENEMQVANGSSSLEKS-YELPDGQVITIADERFRCPEVLFNPAIIGM-EAVGIHETTYNSIMKCDVDIRKD   294
Acetabularia        ERGYSFTTTAEREIVRDIKEKLCYVALDIEQELTS--STETLEKS-YELPDGQVITVGSERFRCPEALFPPGLLGM-EQLGLHDTCFNSIMKCDVDIRKD   291
Amphidinium         ERGYSFTTTAEREIVRDVKEKLCYIALDFDTELKAATESSDKEKT-YELPDGNIITVGAERFRCPEVLFNPSFIGK-EASGIHDTTFQSIMKCDVDIRKD   293
Symbiodinium        ERGYSFTTTAEREIVRDVKEKLCYIALDFDSEMKAASESSDKEKT-YELPDGNIITVGSERFRCPEVLFQPSFVGK-EASGIHDTTFQSIMKCDVDIRKD   293
Lingulodinium       ERGYSFTTTAEREIVRDVKEKLCYIALDYDSEMKAATESSDKEKT-YELPDGNIITVGSERFRCPEVLFQPSLVGK-EASGVHDTTFQSIMKCDVDIRKD   293
Crypthecodinium     ERGYSFTTTAEREIVRDIKEKLSYIALDYDSEMKVATESSDKEKT-YELPDGNIITVGSERFRCPEVLFNPSLLGK-ESSGIHDTTFQSIMKCDVDIRKD   293
Perkinsus           ERGYTFTTTAEREIVRDIKEKLSYVALDFDTEMKAAAESSEKEKT-YELPDGNIITVGNERFRCPEVLFQPTFIGK-EASGIHDTTFQSIMKCDVDIRKD   293
Pythium             ERGYSFTTTAEREIVRDIKEKLTYIALDFDQEMKTAAESSGLEKS-YELPDGNVIVIGNERFRTPEVLFQPSLIGK-EASGIHDCTFQTIMKCDVDIRKD   293
Phytophthora        ERGYSFTTTAEREIVRDIKEKLTYIALDFDQEMKTAAESSGLEKS-YELPDGNVIVIGNERFRTPEVLFQPSLIGK-EASGIHDCTFQTIMKCDVDIRKD   293
Histriculus         ERGYSFTSSAELEIVRDIKEKLCFVALDYEAALKQSHDSSTFEKN-YELPDGKIITIGSERFRCPEYLFKPLEMNGKELDSIQDLTYKSIQECDVDVRRD   292
Oxytricha           ERGYSFTSSAELEIVRDIKEKLCFVALDYEAAYKQSFDSSTFEKN-YELPDGKVITIGSERFRCPEYLFKPLEMNGRELDSIQDLTYKSIQECDVDVRRD   292
Stylonychia         ERGYVFTSSAEMEIVRDIKEXLCFVALDYESALKQSHDSSQFEKN-YELPDGKVITIGNERFRCPEYLFKPLEMNGRELSSIQDLTYNSIQECDVDVRRD   292
Uroleptus           ERGYNFTSSAEMEIVRDIKEKTCFVATDYEAALKQAHDSSTFEKN-YELPDGKVITIGSERFRCPEYLFKPLEMNGRELDSIQDLTYKSIQECDVDVRRE   293
Engelmanniella      ERGYSFTSSAELEIVRDIKEKLCFVALDYEAAMKQSYNSSTFEKN-YELPDGKVITIGNARFRCPEYLFKALEMNGRELDSIQVLTYKSIQECDVDVRRE   293
Hypothrichida       ERGYTFTSSAEMEIVRDIKEKLCFVALDYEAALKQSHDSSTFEKN-YELPDGKVITIGNERFRCPEYXFKPLEMNGRELDSIQSLTYNSIQECDVDVRRD   292
Glamblia            ENGASFTTSAEFEIVRDIKEKLCFVALDYESVLAASMESANYTKT-YELPDGVVITVNQARFKTPELLFRPELNNS-DMDGIHQLCYKTIQKCDIDIRSE   292
Gintestinalis       ENGASFTTSAEFEIVRDIKEKLCFVALDYESVLAASMENTNYTKT-YELPDGVVITVNQARFKTPELLFRPELNNS-DMDGIHQLCYKTIQKCDIDIRSE   292
                    .......210.......220.......230.......240.......250.......260.......270.......280.......290.......300
```

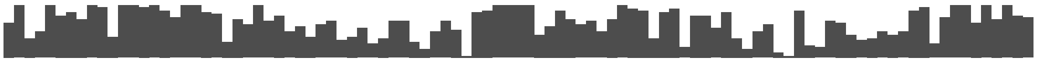

```
                    * . : ***:        *:  ::    *     :: :. :*:: .* **  * **:**    :   :    *.
Tbrucei             LYGNIVLSGGTTMFKNLPERLGKEISNLAPSSIKPKVVAPPERKYSVWIGGSILSSLTTFQSMWITKSEYDESGPSIVHSKCF   376
Tcruzi              LYGNIVLSGGTTMFKNLPERLGKEISNLAPSSIKPKVVAPPERKYSVWIGGSILSSLTTFQTMWIKKSEYDEAGPSIVHNKCF   376
Linfantum           LYGNIVLSGGSTMFLNLPERLAKEISNLAPSSIKPKVVAPPERKYSVWIGGSILSSLTTFQTMWVKKSEYDESGPSIVHNKCF   376
Euglena             LYSNIVLSGGTTMYEGLPERMSKEITNLAPNSMKIKVVAPPERKYSVWIGGSILASLSTFQSMWIKKEEYDEAGPGIVHRKCF   377
Trichomonas         LYANIVLSGGTTMFKGIAERLDKEITALAPPTMKVKIVAPEERKYAVWVGGSILASLATFPQMVITKEEYDEAGPSIVHRKCF   376
Histomonas          LYANIVLSGGTTMFEGLPERMEKEMVPLAPPTMKIKVVAPPERKYAVWIGGSILASLATFPQMVITRDEYNEAGPGIVPRKCF   372
Tetrahymena         PYNNIVLSGGTTMFPGIAERLSKEVSALAPSSMKIKVVAPPERRYSVWIGGSILSSLSTFQTMWITKAEYDESGPSIVHRKCF   376
Paramecium          LYGNVVMSGGTTMFPGIPERLSKELTSLAPSSMKIKVVAPPERKFSVWIGGSILSSLSTFQAMWITRSEYDESGPSIVHRKCF   375
PvivaxII            LYNNIVLSGGTTMYNNIGERLTKEMTNLAPSSMKIKVIAPPERKYSVWIGGSILSSLSTFQQMWITKEEYEDAGPSIVHRKCF   376
PknowlesiII         LYSNIVLSGGTTMYNNIGERLTKEMTNLAPSSMKIKVIAPPERKYSVWIGGSILSSLSTFQQMWITKEEYEDAGPSIVHRKCF   376
PfalciparumII       LYNNIVLSGGTTMYNNIGERLTKEMTNLAPSSMKIKVIAPPERKYSVWIGGSILSSLSTFQQMWITKEEYEDSGPSIVHRKCF   376
PyoeliiII           LYNNIVLSGGTTMYNYIGERLTNEMTNLAPPSMKIKVIAPPERKYSVWIGGSILSSLSTFQKMWITKEEYDESGPSIVHRKCF   376
PbergheiII          LYNNIVLSGGTTMYNYIGERLTNEMTSLAPPSMKIKVIAPPERKYSVWIGGSILSSLSTFQKMWITKEEYDESGPSIVHRKCF   376
Gregarina           LYSNVVLSGGTTMYEGIGERMTKELTDLAPSTVGIKVIAPPERKFSVWIGGSILSSLTTFQNMWITADEYEECGPVIVHKKCM   376
Toxoplasma          LYGNVVLSGGTTMYEGIGERLTKELTSLAPSTMKIKVVAPPERKYSVWIGGSILSSLSTFQQMWITKEEYDESGPSIVHRKCF   376
Neospora            LYGNVVLSGGTTMYEGIGERLTKELTSLAPSTMKIKVVAPPERKYSVWIGGSILSSLSTFQQMWITKEEYDESGPSIVHRKCF   376
Eimeria             LYGNVVLSGGTTMYEGIGERLTRELTSLAPSTMKIKVVAPPERKYSVWIGGSILSSLSTFQQMWITKEEYDESGPSIVHRKCF   374
PvivaxI             LYGNIVLSGGTTMYEGIGERLTRDITTLAPSTMKIKVVAPPERKYSVWIGGSILSSLSTFQQMWITKEEYDESGPSIVHRKCF   376
Pknowlesi           LYGNIVLSGGTTMYEGIGERLTRDITTLAPSTMKIKVVAPPERKYSVWIGGSILSSLSTFQQMWITKEEYDESGPSIVHRKCF   376
PbergheiI           LYGNIVLSGGTTMYEGIGERLTRDITTLAPSTMKIKVVAPLERKYSVWIGGSILSSLSTFQQMWITKEEYDESGPSIVHRKCF   375
Pyoelli             LYGNIVLSGGTTMYEGIGERLTRDITTLAPSTMKIKVVAPPERKYSVWIGGSILSSLSTFQQMWITKEEYDESGPSIVHRKCF   376
PfalciparumI        LYGNIVLSGGTTMYEGTGERLTRDITTLAPSTMKIKVVAPPERKYSVWIGGSILSSLSTFQQMWITKEEYDESGPSIVHRKCF   376
Tparva              LYANVVLSGGTTMFEGIGQRMTKELNALVPSTMKIKVVAPPERKYSVWIGGSILSSLSTFQQMWITKEEFDESGPNIVHRKCF   376
Tannulata           LYANVVLSGGTTMFEGIGQRMTKELNALVPSTMKIKVVAPPERKYSVWIGGSILSSLSTFQQMWITKEEFDESGPNIVHRKCF   376
Babesia             LYANVVLSGGTTMYEGIGQRMTKELNALVPSTMKIKVVAPPERKYSVWIGGSILSSLSTFQQMWITKEEFDESGPNIVHRKCF   376
CparvumACT1         LYANIVLSGGTTMYPGIGERMTKELTSLAPSTMKIKVVAPPERKYSVWIGGSILSSLSTFQQMWITKEEYDESGPSIVHRKCF   376
Chominis            LYANIVLSGGTTMYPGIGERMTKELTSLAPSTMKIKVVAPPERKYSVWIGGSILSSLSTFQQMWITKEEYDESGPSIVHRKCF   376
Cmuris              LYANVVLSGGTTMYPGLGERMTKELTCLAPSTMKIKVVAPPERKYSVWIGGSILSSLSTFQQMWITKEEYDESGPSIVHRKCF   376
Aedes               LYANTVLSGGTTMYPGIADRMQKEITALAPSSIKIKIIAPPERKYSVWIGGSILASLSTFQAMWISKQEYDESGPAIVHRKCF   376
Anopheles           LYANSVLSGGTTMYPGIADRMQKEITSLAPSTIKIKIIAPPERKYSVWIGGSILASLSTFQTMWISKHEYDEGGPGIVHRKCF   376
Drosophila          LYANSVLSGGTTMYPGIADRMQKEITTLAPSTIKIKIIAPPERKYSVWIGGLILASLSTFQQMWISKQEYDESGPSIVHRKCF   376
Culex               LYANTVLSGGTTMYPGIADRMQKEITALAPSTIKIKIIAPPERKYSVWIGGSILASLSTFQAMWISKQEYDESGPSIVHRKCF   376
Ciona               LYANNVLSGGTTMYPGIADRMQKEITALAPSTMKIKIIAPPERKYSVWIGGSILASLSTFQQMWITKQEYDEAGPSIVHRKCF   378
Ornithorhynchus     LYANNVLSGGTTMYPGIADRMQKEITALAPSTMKIKIIAPPERKYSVWIGGSILASLSTFQQMWISKQEYDEAGPSIVHRKCF   377
Strongylocentrotus  LYANTVLSGGTSMYPGIADRMQKEITALAPSSVKIKIIAPPERKYSVWIGGSILASLSTFQQMWISKQEYXXSGASIVHRKCF   376
Homobeta            LYANTVLSGGTTMYPGIADRMQKEITALAPSTMKIKIIAPPERKYSVWIGGSILASLSTFQQMWISKQEYDESGPSIVHRKCF   375
Sus                 LYANTVLSGGTTMYPGIADRMQKEITALAPSTMKIKIIAPPERKYSVWIGGSILASLSTFQQMWISKQEYDESGPSIVHRKCF   375
Gallus              LYANTVLSGGTTMYPGIADRMQKEITALAPSTMKIKIIAPPERRYSVWIGGSILASLSTFQQMWISKQEYDESGPSIVHRKCF   375
Xenopus             LYANTVLSGGTTMYPGIADRMQKEITALAPSTMKIKIIAPPERKYSVWIGGSILASLSTFQQMWISKQEYDESGPSIVHRKCF   375
Monodelphis         LYANTVLSGGTTMYPGIADRMQKEITALAPSTMKIKIIAPPERKYSVWIGGSILASLSTFQQMWISKQEYDESGPSIVHRKCF   376
Danio               LYANTVLSGGTTMYPGIADRMQKEITSLAPSTMKIKIIAPPERKYSVWIGGSILASLSTFQQMWISKQEYDESGPSIVHRKCF   375
Bombyx              LYANTVLSGGTTMYPGIADRMQKEITRLAPSTMKIKIIAPPERKYSVWIGGSILASLSTFQQMWISKQEYDESGPSIVHRKCF   376
Haemaphysalis       LYANTVLSGGTTMYPGIADRMQKEITALAPSTMKIKIIAPPERKYSVWIGGSILASLSTFQQMWISKQEYDESGPSIVHRKCF   376
Caenorhabditis      LYANTVLSGGTTMYPGIADRMQKEITALAPSTMKIKIIAPPERKYSVWIGGSILASLSTFQQMWISKQEYDESGPSIVHRKCF   376
Wuchereria          LYANTVLSGGTSMFPGIADRMQKEITALAPSTMKIKIIAPPERKYSVWIGGSILASLSTFQQMWISKQEYDESGPSIVHRKCF   376
Hydra               LYANTVLSGGTTMFPGIADRMQKEISALAPPTMKIKIIAPPERKYSVWIGGSILASLSTFQQMWISKQEYDESGPSIVHRKCF   376
Haliotis            LYANTVLSGGTTMFPGIADRMQKEITALAPATMKIKIIAPPERKYSVWIGGSILASLSTFQQMWISKQEYDESGPSIVHRKCF   375
Schistosoma         LYSNTVLSGGSTMYPGIADRMQKEISALAPSTMKIKIVAPPERKYSVWIGGSILASLSTFQQMWISKQEYDESGPGIVHRKCF   376
Dictyostelium       LYGNVVLSGGTTMFPGIADRMNKELTALAPSTMKIKIIAPPERKYSVWIGGSILASLSTFQQMWISKEEYDESGPSIVHRKCF   376
Pyrocystis          LYGNVVLSGGSTMFPGIAERMNKELVALAPSTMKIKIIAPPERKYSVWIGGSILASLSTFQQMWISKEEYDESGPSIVHRKCF   375
Thecamoeba          LYGNVVCSGGTTMFPGIADRLQKELTALAPSTMKIKIIAPPERKYSVWIGGSILASLSTFQQMWISKEEYDESGPSIVHRKCF   376
Amoeba              LYGNIVLSGGTTMFPGIADRLQKEIVNLAPSTMKIKIIAPPERKYSVWIGGSILASLSTFQQMWISKEEYDESGPSIVHRKCF   374
Chaos               LYGNIVLSGGTTMFPGIADRLQKEIVNLAPSTMKIKIIAPPERKYSVWIGGSILASLSTFQQMWISKEEYDESGP--------   366
Hartmannella        LYGNIVLSGGTTMFPGIADRLQKELVNLAPSTMKVKIIAPPERKYSVWIGGSILSSLSTFQQMWISKEEYDESGPQIVHRKCF   374
Ehistolytica        LYGNIVLSGGTSMYPGINTRLEKEMIQLAPPTMKIKVIAPPERKYSVWIGGSILASLSTFQNMWITKEEYDESGPAIVHRKCF   376
Edispar             LYGNIVLSGGTSMYPGINTRLEKEMIQLAPPTMKIKVIAPPERKYSVWIGGSILASLSTFQNMWITKEEYDESGPAIVHRKCF   376
Pichia              LYGNIVMSGGTTMFPGIAERMQKEITALAPSSMKVKIIAPPERKYSVWIGGSILASLSTFQQMWISKQEYDESGPSIVHHKCF   376
Candida             LYGNIVMSGGTTMFPGIAERMQKEITALAPSSMKVKIIAPPERKYSVWIGGSILASLSTFQQMWISKQEYDESGPSIVHHKCF   381
Yeast               LYGNIVMSGGTTMFPGIAERMQKEITALAPSSMKVKIIAPPERKYSVWIGGSILASLTTFQQMWISKQEYDESGPSIVHHKCF   375
Nicotinia           LYGNIVLSGGSTMFPGIADRMSKEITALAPSSMKIKVVAPPERKYSVWIGGSILASLSTFQQMWIAKAEYDESGPSIVHRKCF   377
Brachypodium        LYGNIVLSGGTTMFTGIADRMSKEITALAPSSMKIKVVAPPERKYSVWIGGSILASLSTFQQMWIAKAEYDESGPSIVHRKCF   377
Arabidopsis         LYGNIVLSGGTTMFPGIADRMSKEITALAPSSMKIKVVAPPERKYSVWIGGSILASLSTFQQMWIAKAEYDESGPSIVHRKCF   377
Zea                 LYGNIVLSGGTTMFPGIADRMSKEITALAPSSMKIKVVAPPERKYSVWIGGSILASLSTFQQMWISKAEYDESGPAIVHRKCF   377
Physcomitrella      LYGNIVLSGGSTMFPGIADRMSKEITALAPSSMKIKVVAPPERKYSVWIGGSILASLSTFQQMWIAKSEYDESGPSIVHRKCF   377
Dunaliella          LYNNIVLSGGTTMFPGIADRMSKEITALAPSAMKIKVVAPPERKYSVWIGGSILASLSTFQQMWIAKSEYDESGPSIVHRKCF   379
Chlamydomonas       LYNNIVLSGGTTMFPGIADRMSKEITALAPSAMKIKVVAPPERKYSVWIGGSILASLSTFQQMWIAKSEYDESGPSIVHRKCF   377
Micromonas          LYSNIVLSGGTTMFPGVADRMSKEITALAPSSMKIKVVAPPERKYSVWIGGSILASLSTFQQMWIAKSEYDESGPSIVHRKCF   377
Acetabularia        LYSNTVLSGGTMMFPGIGERMQKEITTLAPSSMKIKVVAPPERKFSVWIGGSILSSLSTFQQMWISKEEYDEAGPAIVHRKCF   374
Amphidinium         LYANVVLSGGTTMFPGIGERMTKELTALAPSTMKIKVVAPPERKYSVWIGGSILSSLSTFQQMWISKGEYDESGPTIVHRKCF   376
Symbiodinium        LYANVVLSGGTTMFQGIGERMTKELTALAPSTMKIKVVAPPERKYSVWIGGSILSSLSTFQQMWISKGEYVESGPTIVHRKCF   376
Lingulodinium       LYANVVLSGGTTMFIGIGERMAKELTALAPSTMKIKVVAPPERKYSVWIGGSILSSLSTFQQMWITKTEYDEAGPSIVHRKCF   376
Crypthecodinium     LYANVVLSGGTTMFSGIGERMTKELTALAPSTMKIKVVAPPERKYSVWIGGSILSSLSTFQQMWISKGEYDESGPTIVHRKCF   376
Perkinsus           LYANVVLSGGTTMFNGIGERMTKELTALAPSTMKIKVVAPPERKYSVWIGGSILSSLSTFQQMWISKQEYDESGPTIVHRKCF   376
Pythium             LYCNIVLSGGTTMYPGIGERMTKELTALAPSTMKIKVVAPPERKYSVWIGGSILSSLSTFQQMWISKAEYDESGPSIVHRKCF   376
Phytophthora        LYCNIVLSGGTTMYPGVGERMTKELTALAPSTMKIKVVAPPERKYSVWIGGSILSSLSTFQQMWISKAEYDESGPSIVHRKCF   376
Histriculus         LYQTIILSGGSTSIEGIGERLLKEIENRAPKSINVKVIASPDRRFAVWRGGSTLTSLSTFASMWITKEDYDENGASIVHRKCI   375
Oxytricha           LYQNIILSGGTTMYEGIGERLLKEIENRAPKSINVKVIASPDRRFAVWRGGSTLTSLSTFASMWITKEDYDENGASIVHRKCI   375
Stylonychia         LYQNIILSGGTTMYEGIGERLLKEIENRAPKSITVKVIASPDRRFAVWRGGSTLTSLSTFASMWITKEDYDEHGAQIVHRKCI   375
Uroleptus           LYQNIILSGGTTLYEGIGERLLKEIESRAPKSITVKVIASPDRRFAVWRGGSTLTSLSTFASMWITKEDYDEHGAQIVHRKCI   376
Engelmanniella      LYQNIILSGGTTMYEGTGERLLTEIENRAPKSINVKVIASPDRKYAVWRGGSTLTSLSTFASMWITKEDCDEHGASIVHRKCI   376
Hypothrichida       HYQNIILSGGTTMYEGIGERFLQEIENRAPKSMTVKVIASPDRRFEVWRGGSILSSLSTFATTSITKEEYEENGSHIVHKKCL   375
Glamblia            LYSNVVLSGGSSMFAGLPERLEKELLDLIPAGKRVRISSPEDRKYSAWVGGSVLGSLATFESMWVSSQEYQENGASIANRKCM   375
Gintestinalis       LYSNVVLSGGSSMFTGLPERLEKELLDLIPAGKRVRISSPEDRKYSAWVGGSVLGSLATFESMWVSSQEYQENGASIANRKCM   375
                    .......310.......320.......330.......340.......350.......360.......370.......380...
```

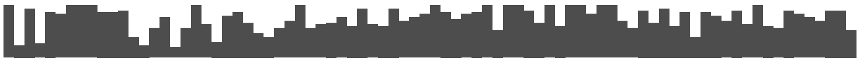

Supplement: Figure S5 — Clustal alignment of actins from a diverse set of protists, fungi, plants, and animals. Maximum likelihood tree was drawn using HyPhy (69) under the HKY85 model with 100 bootstrap replicates. The likelihood for the tree shown is Ln = −12,231. Branches are colored to indicate actins that contain substitutions. Red bars include organisms with K or R at residue 270 (yeast), which is typically hydrophobic and most often L or M in conventional actins (black bars). Green bars indicate organisms that have G at residue 200 (yeast), where most organisms have S, as well as the K270 substitution. (PDF) [file ppat.1002280.s005.pdf]
