# Supplementary material for: Evolutionarily Divergent, Unstable Filamentous Actin Is Essential for Gliding Motility in Apicomplexan Parasites
Source: PLoS Pathog. 2011 Oct 6;7(10):e1002280. doi: 10.1371/journal.ppat.1002280 (PMC3188518; doi:10.1371/journal.ppat.1002280)
Supplement: Table S1 — Primer list for construction of vectors used in current study. (DOCX) [file ppat.1002280.s007.docx]

| Primer | Plasmid | Use |
| --- | --- | --- |
| 5’-CTAGTCTCGAGAATGGGAGAAGAAGTAGTTCAA-3’ (forward)  5’-CTAGTGAGCTCTTAGAAACATTTTCTGTGGACAATAC-3’ (reverse) | pAcHLT-C PfACTI | Expression of recombinant PfACTI with baculovirus |
| 5’-CTAGTCTCGAGGATGTCTGAAGAAGCTGTTG-3’ (forward)  5’-CTAGTGAGCTCTTAGAAACATTTTCTATGAACAATACTAGG-3’ (reverse) | pAcHLT-C PfACTII | Expression of recombinant PfACTII with baculovirus |
| 5’-CTAGTCATATGCATGGATTCTGAGGTTGCT-3’ (forward)  5’-CTAGTGAATTCTTAGAAACACTTGTGGTGAACGAT-3’ (reverse) | pACHLT-C ScACT | Expression of recombinant ScACT with baculovirus |
| 5’-CTCCACGAGAGAGGATACTCCTTCACCACCTCCGCCGAG-3’ (forward)  5’-CTCGGCGGAGGTGGTGAAGGAGTATCCTCTCTCGTGGAG-3’ (reverse | pAcHLT-C TgACTI-G200S and  pAcHLT-C TgACTI-G200S/K270M | Expression of recombinant TgACTI-G200S or TgACTI-  G200S/K270M with baculovirus (site-directed mutagenesis) |
| 5’-AGCCCTCCTTCTTGGGCATGGAGGCTGCAGGTGTCCA-3’ (forward)  5’-TGGACACCTGCAGCCTCCATGCCCAAGAAGGAGGGCT-3’ (reverse) | pAcHLT-C TgACTI-K270M | Expression of recombinant TgACTI-K270M with baculovirus (site-directed mutagenesis) |
| 5’-GCGCCTAGGATGGCGGATGAAGAAGTGCAA-3’ (forward)  5’-CTAGTCTGCAGTTAGAAGCACTTGCGGTGGA-3’ (reverse) | pTUB-DD-myc-TgACTI (WT, G200S or G200S/K270M) | Expression of DD-TgACTI fusion alleles within *Toxoplasma* (Primers add AvrII and PstI sites for cloning) |
| 5’-TTGATATCGAATTCCTTCAGCCCCCGAGACGCGTGTT-3’ (forward)  5’-AACACGCGTCTCGGGGGCTGAAGGAATTCGATATCAA-3’ (reverse) | pTUB-DD-myc-YFP-CAT-minus PstI | One PstI site mutated in pTUB-DD-myc-YFP-CAT in order to have a unique restriction site |

# **Table S1. Primer list for construction of vectors used in current study**
